# Multiplex Neural Circuit Tracing With G-Deleted Rabies Viral Vectors

**DOI:** 10.3389/fncir.2019.00077

**Published:** 2020-01-10

**Authors:** Toshiaki Suzuki, Nao Morimoto, Akinori Akaike, Fumitaka Osakada

**Affiliations:** ^1^Laboratory of Cellular Pharmacology, Graduate School of Pharmaceutical Sciences, Nagoya University, Nagoya, Japan; ^2^Laboratory of Neural Information Processing, Institute for Advanced Research, Nagoya University, Nagoya, Japan; ^3^Institute of Nano-Life-Systems, Institutes of Innovation for Future Society, Nagoya University, Nagoya, Japan; ^4^PRESTO/CREST, Japan Science and Technology Agency, Saitama, Japan

**Keywords:** rabies virus, transsynaptic targeting, neural circuit, multiplex, anatomy

## Abstract

Neural circuits interconnect to organize large-scale networks that generate perception, cognition, memory, and behavior. Information in the nervous system is processed both through parallel, independent circuits and through intermixing circuits. Analyzing the interaction between circuits is particularly indispensable for elucidating how the brain functions. Monosynaptic circuit tracing with glycoprotein (G) gene-deleted rabies viral vectors (RVΔG) comprises a powerful approach for studying the structure and function of neural circuits. Pseudotyping of RVΔG with the foreign envelope EnvA permits expression of transgenes such as fluorescent proteins, genetically-encoded sensors, or optogenetic tools in cells expressing TVA, a cognate receptor for EnvA. Trans-complementation with rabies virus glycoproteins (RV-G) enables trans-synaptic labeling of input neurons directly connected to the starter neurons expressing both TVA and RV-G. However, it remains challenging to simultaneously map neuronal connections from multiple cell populations and their interactions between intermixing circuits solely with the EnvA/TVA-mediated RV tracing system in a single animal. To overcome this limitation, here, we multiplexed RVΔG circuit tracing by optimizing distinct viral envelopes (oEnvX) and their corresponding receptors (oTVX). Based on the EnvB/TVB and EnvE/DR46-TVB systems derived from the avian sarcoma leukosis virus (ASLV), we developed optimized TVB receptors with lower or higher affinity (oTVB-L or oTVB-H) and the chimeric envelope oEnvB, as well as an optimized TVE receptor with higher affinity (oTVE-H) and its chimeric envelope oEnvE. We demonstrated independence of RVΔG infection between the oEnvA/oTVA, oEnvB/oTVB, and oEnvE/oTVE systems and *in vivo* proof-of-concept for multiplex circuit tracing from two distinct classes of layer 5 neurons targeting either other cortical or subcortical areas. We also successfully labeled common input of the lateral geniculate nucleus to both cortico-cortical layer 5 neurons and inhibitory neurons of the mouse V1 with multiplex RVΔG tracing. These oEnvA/oTVA, oEnvB/oTVB, and oEnvE/oTVE systems allow for differential labeling of distinct circuits to uncover the mechanisms underlying parallel processing through independent circuits and integrated processing through interaction between circuits in the brain.

## Introduction

The function of the nervous system arises from complex interactions between networks of neurons composed of multiple cell types. Each cell type has its own molecular, morphological, neurophysiological, and anatomical properties and organizes unique neural circuits. The wiring patterns of individual cell types underlie how neural circuits process and represent information. Detailed information on the cell types and their connectivity, in addition to the spatiotemporal patterns of activity in neural circuits, is essential for understanding how the brain functions. Parallel information processing through independent circuits is a commonly-used strategy in the brain (Nassi and Callaway, 2009), but processed information is combined by integration of neural circuits. Interaction between circuits underlies diverse and complex computations that generate perception, cognition, memory, and behavior. Thus, the combination of parallel processing through independent circuits and integrated processing through interaction between circuits is the fundamental principle of neural circuits and computations in the brain. Despite the advances in methods for linking cell types to neural circuits (Arenkiel and Ehlers, 2009; Luo et al., 2018), a major impediment in elucidating how information processing is integrated in the nervous system is lack of means for the dissection of the complex interactions between neural circuits.

The development of viral vectors has transformed the field of neuroanatomy. In particular, G-deleted rabies viral vectors (RVΔG) have been used to reveal neuronal connectivity in the mammalian nervous system (Wickersham et al., 2007; Marshel et al., 2010; Wall et al., 2010; Osakada et al., 2011; Rancz et al., 2011; Watabe-Uchida et al., 2012; Miyamichi et al., 2013; Osakada and Callaway, 2013; Zhu et al., 2014; Bourane et al., 2015; Wertz et al., 2015; Vaziri and Connor, 2016; François et al., 2017; Sun et al., 2017; Kaelberer et al., 2018). Rabies viruses infect neurons through axon terminals and spread across the synapses between neurons in a retrograde direction (Ugolini, 1995, 2010). To take advantage of their trans-synaptic spread property, cell-type-specific infection of rabies viral vectors is required for mapping the neuronal connectivity of target cell types in the brain. Rabies viruses are enveloped viruses with the rabies virus glycoprotein (RV-G) gene as a native envelope. As the glycoprotein gene is deleted from the viral genome, the envelope of G-deleted rabies viruses can be replaced with a foreign one to change their tropism, so-called pseudotyping (Mebatsion et al., 1996; Etessami et al., 2000; Wickersham et al., 2007). Pseudotyping of RVΔG with a foreign glycoprotein allows viral targeting to specific cell populations that express its receptor. The EnvA/TVA is an avian sarcoma leukosis virus (ASLV) infection system that is not recognized by mammalian cells (Bates et al., 1993; Young et al., 1993; Wickersham et al., 2007). The basic approach for targeting specific cell types is that the initial RV infection is targeted to cells of interest by selectively expressing TVA, a specific receptor for EnvA, in those cells *via* viral vectors, transgenic mice, or *in vivo* electroporation (Wickersham et al., 2007; Marshel et al., 2010; Wall et al., 2010, 2016; Rancz et al., 2011; Watabe-Uchida et al., 2012; Miyamichi et al., 2013; Osakada and Callaway, 2013; Zampieri et al., 2014; Zhu et al., 2014; Kim et al., 2015; Wertz et al., 2015; Faget et al., 2016; Beier et al., 2017; Kaelberer et al., 2018). Co-expression of TVA and rabies glycoprotein RV-G in target neurons referred to as “starter cells,” allows RVΔG to retrogradely spread to presynaptic neurons directly connected to the starter cells.

This RVΔG monosynaptic tracing represents a powerful approach in mice, zebrafish, cats, and non-human primates (Liu et al., 2013; Dohaku et al., 2019). However, several challenges regarding the development of rabies viral vectors still remain. Especially, analyzing the relationships between neural circuits currently presents a substantial bottleneck to revealing the segregation and integration of complex neural circuits. The EnvA/TVA system for rabies viral tracing allows labeling of only single cell types and their presynaptic neurons. Thus, it has been impossible to reveal the structure, function, and interaction of complicated intermixing circuits organized by multiple cell types (Glickfeld et al., 2013; Yamashita et al., 2013; Lur et al., 2016; Kim et al., 2018). In addition, development of multi-colored, genetically-encoded fluorescent proteins, biosensors, and actuators have allowed us to simultaneously observe different populations in real-time (Chen et al., 2013; Marvin et al., 2013, 2019; Dana et al., 2016; Jing et al., 2018; Luo et al., 2018; Patriarchi et al., 2018; Sun et al., 2018; Abdelfattah et al., 2019; Inoue et al., 2019). By incorporating these tools into the RVΔG tracing system, it is possible to directly link circuits and function (Osakada et al., 2011; Wertz et al., 2015; Tian et al., 2016). Visualizing distinct neural populations with their corresponding fluorescent reporters in real-time is a powerful approach for physiological and behavioral studies. To distinguish starter neurons and presynaptic neurons, it is also required to label starter cells with a reporter that differs in color and is included in the expression cassette of TVA and RV-G. In the present study, we aimed to develop new infection systems of RVΔG to reveal circuit structure, function, and interaction of multiple cell types in the brain.

Here, we first introduced new infection systems using EnvB, EnvC, and EnvE that can be used for pseudotyping RVΔG to restrict viral infection to a specific population in the mouse brain. To eliminate pseudo-negative populations as well as cross-infectivity, we further optimized receptors of EnvB, EnvC, and EnvE to simultaneously dissect multiple circuits. We named these optimized infection systems “oEnvX/oTVX systems” (optimized EnvX/TVX). In conjunction with different colors of reporter fluorescence such as GFP, RFP, BFP, and iRFP, and functional fluorescent probes including genetically encoded sensors for Ca^2+^, voltage, and neurotransmitters (Chen et al., 2013; Marvin et al., 2013, 2019; Dana et al., 2016; Jing et al., 2018; Patriarchi et al., 2018; Sun et al., 2018; Abdelfattah et al., 2019; Inoue et al., 2019), the multiplexable virus infection systems will allow for *in vivo* interrogation of complex overlapping neural circuits in a single animal. The present study expands the utility of the RVΔG tracing system to simultaneously label and characterize multiple circuits in single animals. The multiplex RVΔG tracing system promises to advance our understanding of the circuit-level mechanisms underlying information processing in the central nervous system.

## Materials and Methods

### Plasmid Construction

All plasmid construction using polymerase chain reaction (PCR) was performed with PrimeSTAR Max DNA Polymerase (TaKaRa, Tokyo, Japan) on a PCR thermal cycler (Dice Touch, TaKaRa). Plasmids and PCR products were digested with appropriate restriction enzymes (New England BioLabs, Ipswich, MA, USA) at optimal conditions. Plasmid and PCR fragments were assembled with either NEBuilder HiFi DNA Assembly Master Mix (New England BioLabs, Ipswich, MA, USA) or DNA Ligation Kit Mighty Mix (TaKaRa). Chemically-competent Stbl3 *E.coli* (Thermo Fisher Scientific, Waltham, MA, USA) was transformed with AAV plasmids, and chemically-competent XL10-Gold *E. coli* (Agilent Technology, Santa Clara, CA, USA) was used for the other plasmids. The *E. coli* was grown in LB medium (Kanto chemical, Tokyo, Japan) on an LB plate containing ampicillin (100 μg/ml). Plasmids were inspected by diagnostic digestions with appropriate restriction enzymes and sequenced before virus production.

### Cell Culture

HEK293t cells were obtained from RIKEN Cell Bank (Saitama, Japan). HEK-TVA, HEK-TVB, BHK-T7, BHK-EnvA, BHK-EnvB, and B7GG cells were gifted by Dr. Callaway (Salk Institute for Biological Studies, La Jolla, CA, USA; Wickersham et al., 2007; Choi et al., 2010; Osakada et al., 2011; Osakada and Callaway, 2013). These cells were maintained in DMEM (Wako, Osaka, Japan), supplemented with 10% fetal bovine serum (FBS; Sigma, St. Louis, MI, USA), 100 U/ml penicillin G, and 100 μg/ml streptomycin (Wako). Cells were cultured in a humidified atmosphere of 5% CO_2_ and 95% air at 37°C.

### Generation of Cell Lines

HEK-DR46TVB and B7-oEnvE cells were established by the piggyBac system (Ding et al., 2005). pPB-CAG-DR46TVB-IRES-BSD or pPB-CAG-oEnvE-IRES-BSD with pCAG-PBase were co-transfected in HEK293t cells or BHK-T7 cells using Opti-MEM (Thermo Fisher Scientific, Waltham, MA, USA) and Polyethylenimine “Max” (Polyscience Inc., Warrington, PA, USA). Transfected cells were selected in the presence of blasticidin at a concentration of 100 μg/ml (InvivoGen, San Diego, CA, USA). HEK-DR46TVB and B7-oEnvE cells were maintained in DMEM (Wako), supplemented with 10% FBS (Sigma, St. Louis, MI, USA) in a humidified atmosphere of 5% CO_2_ and 95% air at 37°C.

### RVΔG Production

**Figure 1:**
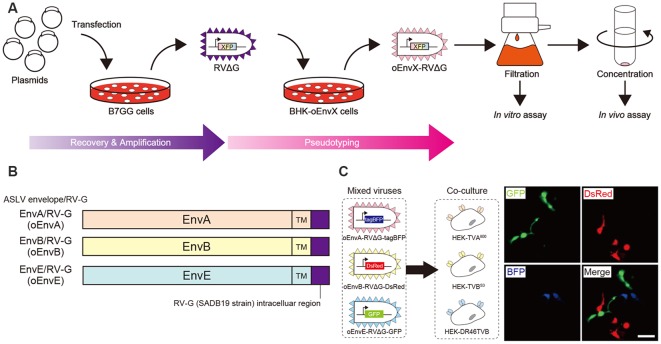
Design and function of avian sarcoma leukosis virus (ASLV)/RV-G chimeric envelope-pseudotyped RVΔG. **(A)** Depiction of oEnvX-pseudotyped RVΔG production. 1st step: recovery of RVΔG viral particles from DNA plasmids and amplification of recovered RVΔG in B7GG cells. 2nd step: generation of RV pseudotyped with oEnvX from oEnvX-expressing cells that were infected with native RVΔG. 3rd step: evaluation of generated viral vectors. The viral supernatant was filtrated for the *in vitro* assay and further concentrated by ultra-centrifugation for the *in vivo* assay. **(B)** Design of the ASLV envelope/RV-G chimeric envelope. oEnvA, oEnvB, and oEnvE were composed of the extracellular and transmembrane domains of EnvA, EnvB, and EnvE envelopes, respectively, and the intracellular domain of RV-G. **(C)** Specific infection of mixed oEnvA-RVΔG-tagBFP, oEnvB-RVΔG-DsRed, and oEnvE-RVΔG-GFP to HEK293t cells expressing their respective receptors TVA^800^, TVB^S3^, and DR46-TVB. Signals were derived from native fluorescence. Scale bar: 50 μm.

RVΔG was produced as described previously (Osakada et al., 2011; Osakada and Callaway, 2013). Briefly, RVΔG-GFP, RVΔG-DsRedexpress, and RVΔG-tagBFP were recovered in B7GG cells by transfection with pcDNA-B19G, pcDNA-B19N, pcDNA-B19P, pcDNA-B19L, and corresponding genomic plasmid (pSADΔG-GFP, pSAD-ΔG-DsRedexpress, or pSAD-ΔG-tagBFP) by Lipofectamine 2000 (Thermo Fisher Scientific, Waltham, MA, USA). The B7GG cells during virus production were cultured in DMEM and supplemented with 10% FBS (Sigma, St. Louis, MI, USA) in a humidified atmosphere of 3% CO_2_ at 35°C. BHK-oEnvA, BHK-oEnvB, or B7-oEnvE cells were infected with recovered viruses for pseudotyping RVΔG with oEnvA, oEnvB, or oEnvE (see also Figure 1B). The virus-containing medium was concentrated by two rounds of ultra-centrifugation (Beckman Coulter, Brea, CA, USA). The infectious titers were determined on HEK-TVA, HEK-TVB, and HEK-DR46TVB cells. HEK293t cells were used to inspect for contamination with unpseudotyped rabies viruses. Virus aliquots were stored at −80°C until use. The titers of the rabies viral vectors used in the present study were 7.0 × 10^5^–2.9 × 10^9^ infectious units/ml.

### AAV Production

AAVs were generated in HEK293t cells with triple-transfection methods with some modifications (Xiao et al., 1998). Briefly, AAV2/2, AAV2/9, and AAV2retro were generated by transfection of HEK293t cells with pHelper (Cell BioLabs, San Diego, CA, USA), the rep/cap vector (pXR2 for AAV2/2, pAAV2.9 for AAV2/9, or rAAV2-retro helper for AAV2retro) and pAAV genomic vector. Three days post-transfection, virus-producing cells were collected and lysed by freeze and thaw for purification. After centrifugation, the supernatant was loaded on gradients (15%, 25%, 40%, and 58%) of iodexanol OptiPrep (Alere Technologies AS, Stirling, Scotland). After centrifugation at 16000 *g* at 15°C for 3 h, 100–150 μl upper from 58% iodexanol layer was collected. Virus aliquots were stored at −80°C until use. The titers of AAV used in the present study were 5.0 × 10^2^ infectious units/ml (HEK293t cells) or 1.0 × 10^11^–1.8 × 10^14^ viral genome/ml quantitative PCR (qPCR; Aurnhammer et al., 2012).

### Animals

Animals were treated in accordance with the Guidelines of Animal Experiments of Nagoya University. All animal experiments in this study were approved by the Animal Care and Use Committee of Nagoya University. All efforts to reduce the number of animals used and minimize the suffering and pain of animals were made. C57BL/6J mice were purchased from Nihon SLC (Shizuoka, Japan). Tlx3-Cre mice (PL56) were obtained from the Jackson Laboratory (Bar Harbor, ME, USA). Genotyping for Tlx3-Cre mice was performed according to the protocol of Mutant Mouse Resource and Research Center: UC Davis. These animals were maintained in a temperature-controlled room (24 ± 1°C) under a 12 h light/dark cycle with* ad libitum* access to food and water.

### Viral Injection

Viral injections were conducted according to the procedures described previously (Osakada et al., 2011) with slight modifications. Briefly, 8-week-old male mice were anesthetized with mixed anesthetic agents (1 ml/kg, i.p.) composed of 75 μg/ml medetomidine hydrochloride (Domitol; Meiji Seika Pharma Company Limited, Tokyo, Japan), 400 μg/ml midazolam (Dormicum; Astellas Pharma Inc., Tokyo, Japan), and 500 μg/ml butorphanol (Vetorphale; Meiji Seika Pharma Company Limited). Anesthetized mice were mounted on a stereotaxic apparatus (David Kopf Instruments, Tujunga, CA, USA). The skull was exposed *via* a small incision, and a small hole was drilled for injection. The virus was loaded into glass pipettes (tip diameter, ≈30 μm; bevel, 45°), pulled on a micropipette puller (P-1000, Sutter Instrument, Novato, CA, USA) and grinded on a microgrinder (EG-401, Narishige, Tokyo, Japan). Before injection of the virus, the pipette was retained in the brain for 5 min after penetration. Three hundered to five hundered nanoliter of virus was injected into the following locations at a rate of 100 nl/min: the V1 (following coordinates: 3.0–3.4 mm rostral, 2.4 mm lateral relative to the bregma and 0.4–0.5 mm ventral to the pia), V2L (3.7 mm rostral, 3.5 mm lateral relative to the bregma and 0.4–0.5 mm ventral to the pia), and SC (3.2 mm rostral, 0.3–0.4 mm lateral relative to the bregma and 2.0 mm ventral to the pia) using a picospritzer (General Valve Corp. Fairfield, NJ, USA). After completion of the injection, the pipette was retained in the brain for another 5 min. No mouse that received viral injection in the brain showed abnormal behavior.

### Histological Analysis

Animals were deeply anesthetized by pentobarbital (Somnopentyl, Kyoritsu Seiyaku Company Limited, Tokyo, Japan) and transcardially perfused with 4% paraformaldehyde in phosphate-buffered saline (PBS). The brains were post-fixed with 4% paraformaldehyde in PBS and cryopreserved overnight in 2% paraformaldehyde/15% sucrose in PBS and then maintained in 30% sucrose in PBS. The brains were sectioned on a freezing microtome (REM-710, Yamato, Saitama, Japan) at 50 μm thickness. For enhancement of native signals, some brain sections were processed for immunostaining as described previously (Osakada et al., 2008, 2011). The primary antibodies and their working dilutions were as follows: rabbit anti-GFP polyclonal (1:2,000, Abcam, UK) and rabbit anti-DsRed polyclonal (1:1,000, Clontech-Takara, Japan). Labeled cells were visualized with the following fluorescent secondary antibodies: anti-mouse IgG and anti-rabbit IgG conjugated with Alexa Flour 488 or Alexa Flour 594 (1:1,000, Jackson Immunoresearch, West Grove, PA, USA). The sections were mounted on slide glasses with anti-fade solution (Nakalai, Kyoto, Japan). Labeled cells were imaged with a confocal laser-scanning microscope with GaAsP detectors (LSM800, Zeiss, Jena, Germany) using a 10× (NA 0.45, Zeiss, Jena, Germany), 20× (NA 0.75, Zeiss, Jena, Germany), or 40× objective lens (NA 1.2, Zeiss, Jena, Germany).

### Statistical Analysis

Data are expressed as means ± SEM. All sets of experiments were performed at least three times. The statistical significance of the difference between groups was determined by one-way ANOVA, followed by Tukey’s or Dunnett’s test using R (R Foundation, Vienna, Austria^1^). Probability values lower than 5% were considered statistically significant.

## Results

### Development of a Chimeric ASLV/RV-G Envelope-Pseudotyped RVΔG

Glycoproteins of enveloped viruses such as rabies viruses recognize and bind to their cell receptors, leading to viral entry. Rabies viruses infect neurons through axon terminals (Ugolini, 1995, 2010). Through replication and transcription in the infected cells, rabies viruses spread across the synapses located between the neurons in the nervous system. Deleting native envelope glycoprotein genes from the rabies genome and pseudotyping the RVΔG with a foreign glycoprotein, such as the EnvA derived from ASLV, allows targeting of viral vectors to specific cell populations that express the receptors of foreign glycoproteins such as TVA (Mebatsion et al., 1996; Etessami et al., 2000; Wickersham et al., 2007; Osakada and Callaway, 2013; Figure 1A). Notably, ASLV uses various other viral envelopes such as EnvB, C, D, E, and J, in addition to EnvA (Barnard and Young, 2003; Barnard et al., 2006). The cloning of ASLV envelopes and their receptors has revealed their specificity (Barnard et al., 2006; Chai and Bates, 2006): TVB^S1^ for EnvB and EnvE, TVB^S3^ for EnvB, TVC for EnvC, and TVJ for EnvJ (Smith et al., 1998; Adkins et al., 2000; Elleder et al., 2005; Chai and Bates, 2006). By using these systems, lentiviral vectors pseudotyped with EnvB, EnvC, and EnvE have been reported to infect cells expressing their corresponding receptors (Matsuyama et al., 2015).

To take advantage of this system to target rabies viral vectors to a particular cell population, RVΔG needs to be pseudotyped with the foreign ASLV envelopes. However, ASLV native envelope proteins are optimized to ASLV but not to rabies viruses. Thus, engineering envelope proteins is required for packaging the rabies viral core with the foreign envelopes. First, we designed chimeric glycoproteins because the native EnvXs envelopes are not compatible with rabies viral cores. Previous reports have used the EnvA/RV-G chimeric protein consisting of an extracellular domain of EnvA and an intracellular domain of rabies viral glycoproteins for RVΔG pseudotyping (Wickersham et al., 2007; Osakada and Callaway, 2013). According to the EnvA/RV-G design, we replaced the intracellular domain of EnvB, EnvC, or EnvE with that of the rabies viral glycoprotein RV-G to generate EnvB/RV-G, EnvC/RV-G, or EnvE/RV-G, respectively. To differentiate the chimeric glycoproteins (EnvX/RV-G) for RVΔG from the native glycoproteins (EnvX) for ASLV, we termed the optimized chimeric glycoproteins EnvX/RV-G “oEnvX” (i.e., EnvA/RV-G for oEnvA, EnvB/RV-G for oEnvB, EnvC/RV-G for oEnvC, and EnvE/RV-G for oEnvE; Figure 1B). EnvD was excluded from this procedure because the EnvD-packaged virus may infect mammalian cells that lack foreign receptors (Bova et al., 1988). EnvJ was also omitted because TVJ, an EnvJ receptor, is a sodium-proton exchanger that may potentially affect neuronal activity (Chai and Bates, 2006; Matsuyama et al., 2015).

Using these chimeric envelopes, we next produced pseudotyped RVΔG with oEnvB, oEnvC, or oEnvE, by transiently expressing them in BHK cells by plasmid transfection. First, we determined whether these pseudotyped RVΔG can infect the mammalian HEK293t cells without introducing the ASLV receptors. No infection with oEnvB- or oEnvE-pseudotyped RVΔG was observed in HEK293t cells (Table 1). However, we found a degree of infection with oEnvC-pseudotyped RVΔG in HEK293t cells (data not shown), suggesting non-specific infection in mammalian cells.

**Table 1:** No cross-infectivity of oEnvA-RVΔG, oEnvB-RVΔG, and oEnvE-RVΔG.

	Infectious Units (/ml)			
Virus Name	HEK-TVA^800^	HEK-TVB^S3^	HEK-DR46-TVB	HEK293t
oEnvA-RVΔG-DsRed	5.3 × 10^7^	n.d.	n.d.	n.d.
oEnvB-RVΔG-GFP	n.d.	4.2 × 10^6^	n.d.	n.d.
oEnvE-RVΔG-DsRed	n.d.	n.d.	7.0 × 10^5^	n.d.

*HEK cells expressing either TVA, TVB, or DR46TVB were respectively infected with oEnvA-RVΔG, oEnvB-RVΔG, or oEnvE-RVΔG. No infections with oEnvA-RVΔG, oEnvB-RVΔG, and oEnvE-RVΔG were observed in HEK293t cells. Data represent the infectious units. n.d.: not detected*.

Thus, we excluded the oEnvC/TVC system from the following studies. To test for the specificity of the pseudotyped RVΔG, we determined whether a mixture of oEnvA-, oEnvB-, and oEnvE-pseudotyped RVΔGs could cause cross-infection to a coculture of TVA^800^-, TVB^S3^-, and DR46-TVB-expressing cells. To distinguish viral infection in three independent cell groups in a mixed population, we prepared pseudotyped RVΔG with distinct envelopes that encode different fluorescent proteins: oEnvA-RVΔG-tagBFP (blue), oEnvB-RVΔG-DsRed (red), and oEnvE-RVΔG-GFP (green; Figure 1C). We applied the mixture of the viruses to the mixed culture of those TVX-expressing cells. Three days after viral infection, the infected cells expressed only one fluorescent protein. No cells expressed multiple colored fluorescent proteins. Therefore, we conclude that oEnvA-, oEnvB-, and oEnvE-RVΔG specifically infect cells expressing TVA^800^, TVB^S3^, and DR46-TVB, respectively. These results imply that oEnvA/TVA, oEnvB/TVB, and oEnvE/DR46-TVB can be applied to multiplexing RVΔG infection.

To apply the multiplex infection system *in vivo*, we need to obtain the pseudotyped RVΔG at high titers. We next attempted to establish an efficient virus-production system. Since cell lines expressing EnvA/RV-G or EnvB/RV-G generated high titers of the pseudotyped RVΔG (Wickersham et al., 2007; Choi et al., 2010; Osakada et al., 2011), we also generated an oEnvE-expressing cell line. Generally, the efficiency of RVΔG pseudotyping depends on the expression level of the envelope of the host cell. Therefore, to establish a cell line expressing an envelope protein at a higher expression level, we used the piggyBac transposon system that enables integrating multiple copies of exogenous genes into the chromosomal DNA of the host cells (Ding et al., 2005). We successfully generated BHK-T7 cells stably expressing oEnvE and named them B7-oEnvE cells. Using these oEnvX-cell lines, we successfully produced pseudotyped RVΔG at a high titer: oEnvA-RVΔG at 5.3 × 10^7^, oEnvB-RVΔG at 4.2 × 10^6^, and oEnvE-RVΔG at 7.0 × 10^5^ (Table 1). These results suggest that the stable cell lines expressing chimeric envelopes can efficiently generate high titers of pseudotyped RVΔG that can be used for *in vivo* experiments.

### Generation of the Optimized oEnvA/oTVA System

For multiplex RVΔG tracing, we need to reliably visualize starter neurons expressing TVX receptors. For this purpose, we also envisioned to functionally characterize the connectionally defined neurons by combining RVΔG trans-synaptic tracing with *in vivo* functional imaging. To reveal the relationship between neuronal connectivity and function, we used fluorescent indicators for calcium, voltage, or neurotransmitters together with the RVΔG system for *in vivo* functional imaging. The hurdle of such an experiment is that co-labeling of the connectionally defined neurons with fluorescent reporters (green or red) and indicators is not useful, as many fluorescent indicators such as GCaMP, XCaMPs, jRGECO, iGluRSnFr, and dLight1 are of the same color (Chen et al., 2013; Marvin et al., 2013, 2019; Dana et al., 2016; Jing et al., 2018; Patriarchi et al., 2018; Sun et al., 2018; Inoue et al., 2019). Thus, infrared fluorescent reporters that do not interfere with green and red fluorescence are required for co-labeling of the connectionally defined neurons (Shcherbakova and Verkhusha, 2013). Therefore, to simultaneously visualize multiple circuits with different colors, we generated an infrared-tagged TVA receptor for starter neuron labeling.

TVA has variants with different affinities. Among them, the original type TVA^800^ displays the highest affinity to EnvA; however, TVA^800^ is a glycophosphatidylinositol-anchored receptor without a transmembrane region (Bates et al., 1993; Gray et al., 2011). Thus, its N-terminal cannot be fused to a fluorescent protein for its visualization. Conversely, TVA^950^ is a single transmembrane receptor and can be fused to a fluorescent protein for the visualization of TVA-expressing starter cells: TVA^950^-EYFP (Faget et al., 2016), TVA^950^-mCherry (Watabe-Uchida et al., 2012), and low-affinity mutant TVA^950E66T^-mCherry (Miyamichi et al., 2013). The high sensitivity of TVA^950^-mCherry causes oEnvA-RVΔG-GFP infection to cells expressing TVA^950^-mCherry at a level where mCherry signals are not detected. Using TVA^950^-mCherry for rabies trans-complementation experiments, the starter cells appear mCherry-negative and GFP-positive with subsequent pseudo-negative results. To overcome this issue, we utilized TVA^950E66T^, a mutant TVA at E66T, with a lower affinity to oEnvA-RVΔG viruses (Rong et al., 1998; Miyamichi et al., 2013). Accordingly, the cells expressing TVA^950E66T^-mCherry at higher levels can be infected with oEnvA-RVΔG viruses allowing elimination of pseudo-negative cell populations because starter cells expressing high levels of mCherry can be unambiguously observed. Cells expressing lower levels of TVA^950E66T^ with undetectable mCherry cannot be infected with oEnvA-RVΔG viruses. The strength of TVA^950E66T^-mCherry is the clear and reliable visualization of starter neurons during rabies tracing experiments because a high TVA^950E66T^-mCherry expression is required for oEnvA-RVΔG infection.

**Figure 2:**
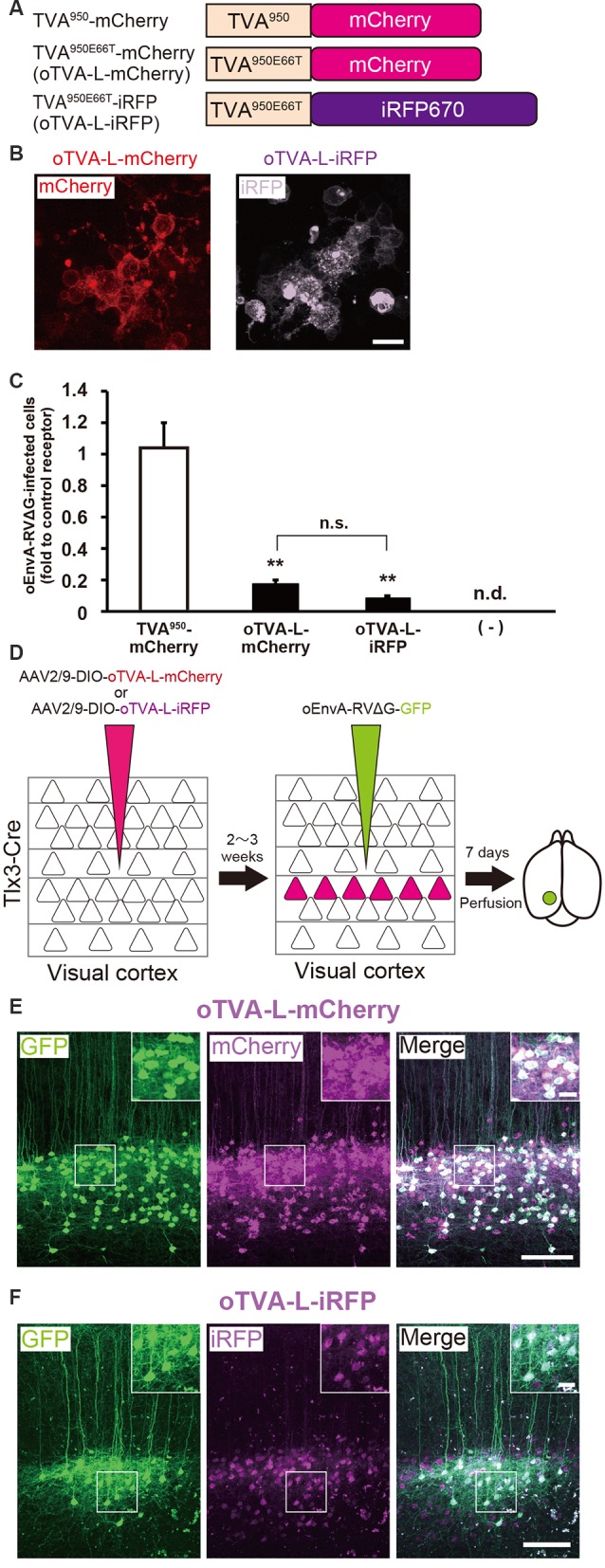
Generation of an optimized oEnvA/oTVA system for unambiguously marking starter cells. **(A)** Design of TVA variants fused to a fluorescent protein. TVA^950^ and its lower-affinity version TVA^950E66T^ (oTVA-L) were fused to mCherry or iRFP670. **(B)** Membrane localization of oTVA-L-mCherry (left) and oTVA-L-iRFP (right) in transfected HEK293t cells. Scale bar: 20 μm. **(C)** Infection efficiency of oEnvA-RVΔG to cells expressing TVA^950^-mCherry, oTVA-L-mCherry, or oTVA-L-iRFP. Each column represents the mean ± SEM (*n* = 3). **P < 0.01 vs. TVA^950^-mCherry (Tukey’s multiple tests). n.s.: not significant. n.d.: not detected. **(D)** *In vivo* evaluation of oTVA-L-iRFP function in the mouse brain. AAV2/9-DIO-oTVA-L-mCherry or AAV2/9-DIO-oTVA-L-iRFP was injected to the V1 of layer 5-specific Tlx3-Cre mice to introduce oTVA-L-mCherry or oTVA-L-iRFP in layer 5 neurons. oEnvA-RVΔG-GFP was injected at the location of the AAV injection. **(E)** Reliable targeting of oEnvA-RVΔG-GFP to oTVA-L-mCherry-expressing cells. oTVA-L-mCherry was expressed in layer 5 neurons in the V1 of Tlx3-Cre mice. All oEnvA-RVΔG-GFP-infected neurons expressed oTVA-L-mCherry. Signals were derived from native fluorescence. Scale bar: 100 μm. The inset shows high magnifications of areas indicated by the white square. Scale bar: 20 μm. **(F)** Reliable targeting of oEnvA-RVΔG-GFP to oTVA-L-iRFP670-expressing cells. oTVA-L-iRFP was expressed in layer 5 neurons in the V1 of Tlx3-Cre mice. All oEnvA-RVΔG-GFP-infected neurons expressed oTVA-L-iRFP. Signals were derived from native fluorescence. Scale bar: 100 μm. The inset shows high magnifications of areas indicated by the white square. Scale bar: 20 μm.

To develop a new TVA that could be used together with green and red fluorescence, we generated TVA^950E66T^ fused with iRFP670 (termed “oTVA-L-iRFP”) as a reporter, the brightest infrared fluorescent protein at present (Shcherbakova and Verkhusha, 2013; Shcherbakova et al., 2018; Figure 2A). First, to examine the subcellular localization of oTVA-L-iRFP, the encoding plasmid was transfected into HEK293t cells (Figure 2B). Confocal microscopy revealed that the iRFP670 signals derived from oTVA-L were localized on the cell membrane, consistent with TVA^950E66T^-mCherry membrane localization. We next investigated whether oEnvA-RVΔG could infect the oTVA-L-iRFP-expressing cells (Figure 2C). oEnvA-RVΔG-GFP was applied to the oTVA-L-iRFP-transfected HEK293t cells. Most iRFP^+^ cells were also GFP-positive. While the TVA^950^-expressing cells were efficiently infected with oEnvA-RVΔG, infection of oTVA-L-iRFP-expressing cells was comparable to that of oTVA-L-mCherry-expressing cells. These results indicate that oTVA-L-iRFP can be used as a receptor for oEnvA-RVΔG *in vitro*, similar to oTVA-L-mCherry.

We next evaluated the *in vivo* function of oTVA-L-mCherry and oTVA-L-iRFP to determine whether they could be used for rabies tracing experiments in the mouse brain. To introduce oTVA-L to a specific population in the brain, we used Tlx3-Cre mice, in which layer 5 cortical neurons specifically express Cre recombinase (Kim et al., 2015; Figure 2D). AAV2/9-CAG-DIO-oTVA-mCherry or AAV2/9-CAG-DIO-oTVA-L-iRFP that expresses oTVA-L-mCherry or oTVA-L-iRFP in a Cre-dependent manner, respectively was used as control. We injected AAV2/9-CAG-DIO-oTVA-L-mCherry or AAV2/9-CAG-DIO-oTVA-L-iRFP into the primary visual cortex (V1) of Tlx3-Cre mice and then oEnvA-RVΔG-GFP in the AAV-injection location (Figure 2D). Seven days after oEnvA-RVΔG injection, we fixed and sectioned the brain of the virus-injected mice. The signal of mCherry was specifically observed in layer 5 neurons around the V1 injection site (Figure 2E), indicating that oTVA-L was expressed in layer 5 neurons in a Cre-dependent manner. Part of the mCherry^+^ cells expressed GFP in layer 5. All GFP^+^ cells were also mCherry-positive in layer 5, and none of the GFP^+^ cells were distributed in the other V1 layers (total 444 mCherry-expressing neurons/444 GFP-expressing neurons from three mice). These results indicate that oEnvA-RVΔG-GFP specifically infected the oTVA-L-mCherry-expressing neurons in layer 5 of the mouse cortex.

Similar results were obtained for oTVA-L-iRFP (Figure 2F). iRFP-positive cells were localized in layer 5 of the V1. Part of the iRFP^+^ cells expressed GFP in layer 5. All GFP^+^ cells were positive for iRFP670 in layer 5 (total 261 iRFP-expressing neurons/261 GFP-expressing neurons from three mice). These results indicate that oEnvA-RVΔG-GFP specifically infected the oTVA-L-iRFP-expressing neurons *in vivo*. No infection with oEnvA-RVΔG-DsRed was observed in the absence of oTVA-L in mice (Supplementary Figure S1A). Thus, the initially-infected cells can be reliably visualized with oTVA-L-iRFP *in vivo*, similar to oTVA-L-mCherry (Figures 2E,F).

To determine the specificity and cross-infectivity of oTVA-L to EnvX-RVΔG, we applied each oEnvX-pseudotyped RVΔG to oTVA-L-expressing HEK293t cells and quantified the number of oEnvX-RVΔG-infected cells (Table 2). oEnvA-RVΔG infected the oTVA-L-expressing cells, whereas oEnvB-RVΔG and oEnvE-RVΔG did not. Taking together, we conclude that oTVA-L is specific to oEnvA-RVΔG and that its fusion reporter allows reliable visualization of the oTVA-L-expressing starter cells *in vivo*.

**Table 2:** Specificity of oTVA-L, oTVB-L, and oTVE-H.

	Number of Infected Cells			
Chimeric Envelope	oTVA-L (TVA^950E66T^)	oTVB-H (TVB^S3^-TVA)	oTVE-H (DR46-TVB-TVA)	(−)
oEnvA-RVΔG	119 ± 8.5	n.d.	n.d.	n.d.
oEnvB-RVΔG	n.d.	112 ± 11	n.d.	n.d.
oEnvE-RVΔG	n.d.	n.d.	>200	n.d.

*HEK cells expressing either oTVA-L, oTVB-L, and oTVE-H were specifically infected with oEnvA-RVΔG, oEnvB-RVΔG, or oEnvE-RVΔG. oTVA-L, oTVB-L, and oTVE-H were specific to oEnvA-RVΔG, oEnvB-RVΔG, and oEnvE-RVΔG, respectively. No infections with oEnvA-RVΔG, oEnvB-RVΔG, and oEnvE-RVΔG were observed in HEK293t cells. Data represent the infectious units. n.d.: not detected*.

### Generation of the Optimized oEnvB/oTVB System

EnvA/TVA allows viral targeting to a particular cell population. To target two distinct populations with two different viruses, we next designed an additional infection system based on the EnvB/TVB system. oEnvB-pseudotyped rabies viral vectors have the potential to infect *via* TVB *in vivo* (Choi et al., 2010). We first attempted to develop TVB, a receptor for oEnvB-enveloped viruses. As the original TVB has several use issues, we optimized the TVB receptors for the rabies tracing experiments focusing on envelope affinity and fluorescence reporter detectivity to utilize the EnvB/TVB system for restricted viral transduction in a specific neuronal population.

**Figure 3:**
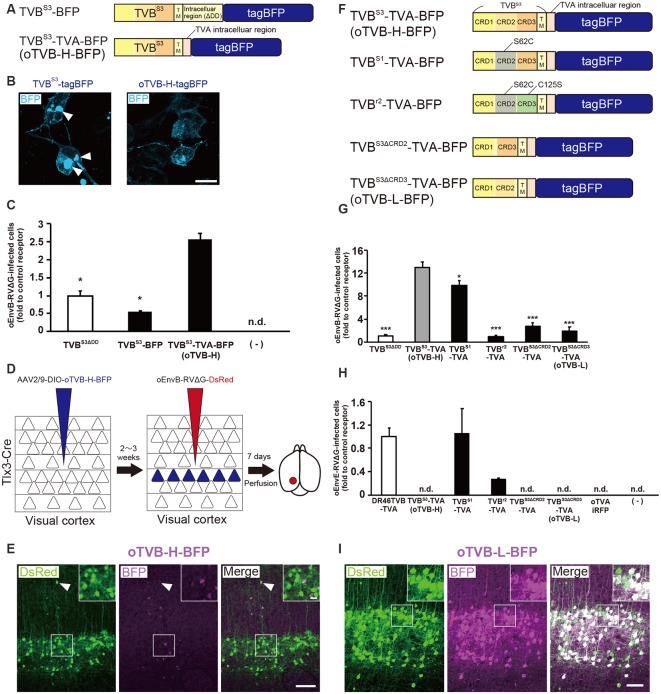
Generation of an optimized oEnvB/oTVB system for viral targeting. **(A)** Design of TVB variants fused to a blue fluorescent protein. TVB^S3ΔDD^-BFP was deficient in the death domain from TVB^S3^ and fused to tagBFP. TVB^S3ΔDD^-TVA-BFP (oTVB-H) has a TVA^950^ intracellular region instead of the TVB^S3^ intracellular region and fused-tagBFP. **(B)** Subcellular localization of TVB^S3^-BFP (left) and oTVB-H-BFP (right) in transfected-HEK293t cells. Abnormal localization of TVB^S3^-BFP in the cell membrane and cytoplasmic region. Arrowhead: aggregation of TVB^S3^-BFP. Membrane localization of oTVB-H-BFP. Scale bar: 20 μm. **(C)** Infectious efficiency of oEnvB-RVΔG to cells expressing TVB^S3ΔDD^, TVB^S3^-BFP, or oTVB-H-BFP.Each column represents the mean ± SEM (*n* = 3). *P < 0.05 vs. oTVB-H (Tukey’s multiple tests). n.d.; not detected. **(D)** *In vivo* evaluation of oTVB-H function in the mouse brain. AAV2/9-DIO-oTVB-H-BFP was injected to the V1 of Tlx3-Cre mice to introduce oTVB-H in layer 5 neurons. oEnvA-RVΔG-DsRed was injected at the location of the AAV injection. **(E)** Evaluation of *in vivo* function of TVB^S3^-TVA-BFP (oTVB-H) in Tlx3-Cre mice. oEnvB-RVΔG-DsRed infected layer 5 neurons without visible oTVB-H-tagBFP signals, suggesting that oTVB-H was sufficiently sensitive for oEnvB-RVΔG-DsRed to infect cells expressing oTVB-H at a low expression level. The arrowhead indicates non-specific infection to neurons in layer 2/3. Signals were derived from native fluorescence. Scale bar: 100 μm. The inset shows high magnifications of areas indicated by the white square. Scale bar: 20 μm. **(F)** Generation of TVB mutants fused with a blue fluorescent protein. TVB^S1^ has one mutation (S62C). TVB^r2^ has two mutations (S62C and C125S). TVB^S3ΔCRD2^ is devoid of CRD2 from TVB^S3^. TVB^S3ΔCRD3^ is devoid of CRD3 from TVB^S3^. **(G,H)** Infection efficiency of **(G)** oEnvB-RVΔG and **(H)** oEnvE-RVΔG to cells expressing one of the tagBFP-fused TVB mutants. Each column represents the mean ± SEM (*n* = 3). *P < 0.05, ***P < 0.001 vs. TVB^S3^-TVA-BFP (oTVB-H) (Dunnett’s multiple test). **(I)** Evaluation of *in vivo* function of TVB^S3ΔCRD3^-TVA-BFP (oTVB-L). oEnvB-RVΔG-DsRed infected oTVB-L-BFP-expressing neurons of layer 5-specific Tlx3-Cre mice. Signals were derived from native fluorescence. Scale bar: 100 μm. The inset shows high magnifications of areas indicated by the white square. Scale bar: 20 μm.

TVB reportedly has the following variants with different affinities for various ASLVs: TVB^S1^, TVB^r2^, TVB^S3^, and TVB^T^ (Brojatsch et al., 1996; Adkins et al., 1997, 2000; Smith et al., 1998). Among the several subtypes, we selected TVB^S3^ because it does not recognize and bind to EnvE, whereas the other TVB variants bind to both EnvB and EnvE (Adkins et al., 2000; Klucking and Young, 2004; Reinisová et al., 2008; Matsuyama et al., 2015). However, TVB is a homolog of the mammalian TNF-related apoptosis-inducing ligand death receptor 4/5; when ASLV-B and ASLV-E infected cells *via* TVB, TVB induced apoptosis (Brojatsch et al., 1996, 2000; Smith et al., 1998; Klucking et al., 2005). The death domain of TVB has been reported to be responsible for inducing apoptosis (Adkins et al., 2000). To maintain the infected neurons healthy during the viral tracing experiments, we deleted the death domain from the full-length of the TVB receptor. Additionally, to visualize the TVB-expressing cells, we replaced the TVB^S3^ death domain with tagBFP, the brightest fluorescent blue protein (Figure 3A). The tagBFP signal was weakly observed at the cell membrane and strongly at the cytoplasm of HEK293t cells transfected with a TVB^S3^-tagBFP plasmid (Figure 3B). Cytoplasm aggregation could cause cell cytotoxicity (Eisele et al., 2015; Boland et al., 2018). Thus, to reduce TVB^S3^-tagBFP aggregation, we designed a new TVB receptor. First, we attempted to insert a GGG peptide linker as a fusion protein, such as TVA^950^-mCherry, into the TVB^S3^-tagBFP segment. However, no change was shown (data not shown). As TVA^950^ was distributed on the membrane (Figure 2B), we replaced the TVB cytoplasmic tail with TVA^950^ to generate a TVB^S3^-TVA chimera receptor (Figure 3A, Supplementary Figure S2). The TVB^S3^-TVA chimera receptor was fused to tagBFP and expressed in the cell membrane without aggregating inside the cell (Figure 3B). To determine whether the cells expressing these TVB variants could be infected with oEnvB-RVΔG, we transfected HEK293t cells with TVB^S3^-TVA-tagBFP and then we applied oEnvB-RVΔG-DsRed to the transfected cells (Figure 3C). Three days after the viral infection, TVB^S3^-TVA-tagBFP-expressing cells were positive for DsRed. TVB^S3^-TVA-tagBFP had higher infection efficiency by oEnvB-RVΔG compared to TVB^S3^-tagBFP. We termed the TVB^S3^-TVA receptor “oTVB-H” (optimized TVB with the highest affinity). We further assessed the cross-infectivity of oTVB-H to oEnvX-pseudotyped RVΔG. Each oEnvX-pseudotyped RVΔG was applied to oTVB-H-expressing HEK293t cells (Table 2). The oEnvB-RVΔG infected the oTVB-H-expressing cells whereas oEnvA-RVΔG and oEnvE-RVΔG did not. Taken together, these results indicate that oTVB-H is a high-affinity receptor with a specificity to oEnvB-RVΔG.

We next investigated whether oTVB-H can be used for viral targeting of a specific population in the brain. To restrict the viral infection to a particular cell population, we again used Tlx3-Cre mice, a layer 5-specific Cre-line; we injected Cre-dependent AAV expressing oTVB-H-tagBFP (AAV2/9-CAG-DIO-oTVB-H-tagBFP) to the V1 of Tlx3-Cre mice (Figure 3D). oEnvB-RVΔG-DsRed was injected at the same location in the V1 2–3 weeks after the AAV injection. The DsRed signal was observed in layer 5 neurons in Tlx3-Cre mice, but not in the neurons or glia of other layers; however, the DsRed^+^ cells in layer 5 were tagBFP-negative (Figure 3E). Tlx3-Cre mice that received injections of oEnvB-RVΔG-DsRed but not of AAV2/9-CAG-DIO-oTVB-H-tagBFP were used as controls, and as expected, no DsRed^+^ cells were observed in the absence of oTVB-H. Our results suggest that the oEnvB-RVΔG-DsRed-infected layer 5 neurons express oTVB-H in a Cre-dependent manner, probably because oTVB-H is markedly sensitive to oEnvB-RVΔG infection; even when the BFP reporter signal cannot be detected due to the low expression level of oTVB-H-BFP, oEnvB-RVΔG can still infect these cells.

To overcome this issue, we attempted to clearly visualize the starter cells with fluorescent reporters by developing a low-affinity type TVB receptor, as shown in the cases of TVA^950^ and TVA^950E66T^ (Watabe-Uchida et al., 2012; Miyamichi et al., 2013; Figure 2). To obtain an ideal receptor, we compared it with other TVB subtypes (Figure 3F). TVB has four subtypes (TVB^S1^, TVB^r2^, TVB^S3^, and TVB^T^) that can recognize and bind to ASLV-B (Brojatsch et al., 1996; Reinisová et al., 2008). The extracellular domain of TVB is composed of three cysteine-rich domains (CRDs; Hymowitz et al., 1999; Adkins et al., 2000; Reinisová et al., 2008). In general, CRD1 is essential for ASLV-B recognition, but CRD2 and CRD3 are not necessary for ASLV-B interaction. Importantly, when these CRD2 and CRD3 domains were mutated or deleted in TVB, ASLV-B infection efficiency tended to decrease (Adkins et al., 2001; Klucking and Young, 2004). Thus, we hypothesized that we can design TVB receptors with lower affinity and higher specificity to EnvB by mutations or deletions of CRD2 and CRD3 from the TVB extracellular domain. Consequently, we designed four TVB mutants that contained a TVA intracellular domain: TVB^S1^-TVA-tagBFP, TVB^r2^-TVA-tagBFP, TVB^S3ΔCRD2^-TVA-tagBFP, and TVB^S3ΔCRD3^-TVA-tagBFP (Figure 3F). Compared to TVB^S3^, TVB^S1^ has only one mutation (S62C) at CRD2, and TVB^r2^ has two mutations at CRD2 (S62C) and CRD3 (C125S; Reinisová et al., 2008). Previous reports have revealed that TVB^S1^ whose CRD2 or CRD3 was deleted showed reduced ASLV-E and stable or slightly reduced ASLV-B infectivity (Adkins et al., 2001; Klucking and Young, 2004). Thus, we expected that chimeric TVB^r2^ or TVB^S3^ without CRD2 or CRD3 would have a lower affinity and higher specificity to EnvB. Accordingly, we generated a CRD2-deleted mutant TVB^S3ΔCRD2^ and a CRD3-deleted mutant TVB^S3ΔCRD3^ (Supplementary Figure S2). To evaluate the *in vitro* localization and function of these TVB mutant receptors, we transfected HEK293t cells with plasmids coding for those mutant receptors and then applied oEnvB-RVΔG-DsRed or oEnvE-RVΔG-GFP to those cells (Figures 3G,H) because several lines of evidence have indicated that TVB^S1^ and TVB^T^ have affinities to ASLV-B and ASLV-E (Brojatsch et al., 2000; Klucking and Young, 2004; Matsuyama et al., 2015). We counted the number of infected cells 3 days after the infection. The cells expressing each TVB mutant receptor were infected with oEnvB-RVΔG-DsRed at a different efficiency (Figure 3G). The oTVB-H-expressing cells were the most efficiently infected with oEnvB-RVΔG-DsRed but did not show infectivity with oEnvE-RVΔG-GFP (Figures 3G,H). TVB^r2^-TVA showed the lowest affinity to oEnvB-RVΔG and limited affinity to oEnvE-RVΔG. Notably, HEK293t cells expressing DR46TVB-TVA, TVB^S1^-TVA, and TVB^r2^-TVA were infected with oEnvE-RVΔG, but no infection with oEnvE-RVΔG was observed in HEK293t cells expressing TVB^S3ΔCRD2^-TVA, TVB^S3ΔCRD3^-TVA, or oTVA-iRFP (Figure 3H). TVB^S3ΔCRD2^-TVA-tagBFP and TVB^S3ΔCRD3^-TVA-tagBFP had the lowest affinity and highest specificity to oEnvB-RVΔG but not to oEnvE-RVΔG. These results suggest that TVB^S3ΔCRD3^-TVA can be used as a specific TVB receptor with the lowest affinity to oEnvB-RVΔG. Hereafter, we refer to TVB^S3ΔCRD3^-TVA as “oTVB-L” (optimized TVB with the lowest affinity).

Then, we tested whether oTVB-L-tagBFP can be used for *in vivo* viral targeting to specific cell types and unambiguous visualization of the infected cells. We injected Cre-dependent AAV expressing oTVB-L-tagBFP (AAV2/9-CAG-DIO-oTVB-L-tagBFP) into the V1 of Tlx3-Cre mice to restrict oTVB-L-tagBFP expression to layer 5 neurons (Figure 3I). Three weeks after injection, the tagBFP signal was distributed in layer 5 neurons of the V1. We subsequently examined whether oEnvB-RVΔG could infect oTVB-L-expressing neurons *in vivo* and whether the oTVB-L-expressing cells could be visible and unambiguously identified as starter neurons for rabies tracing experiments. We injected AAV2/9-CAG-DIO-oTVB-L-tagBFP into the V1 of Tlx3-Cre mice, and then oEnvB-RVΔG-DsRed into the same location. BFP signals were observed in layer 5 neurons of the mouse V1. A total of 84.8 ± 4.2% of DsRed^+^ cells co-expressed tagBFP (total 325/347 neurons from four mice; Figure 3I). Conversely, no infection with oEnvB-RVΔG-DsRed was observed in the absence of oTVB-L in mice that were not subjected to injection of oTVB-L-tagBFP-expressing AAV (Supplementary Figure S1B). These results indicate that oEnvB-RVΔG infected the oTVB-L-expressing neurons and that oTVB-L-tagBFP reliably visualized the oEnvB-RVΔG-infected neurons. Moreover, oTVB-H is too sensitive for use in *in* *vivo* viral targeting because of its low expression level that can lead to viral infection, thereby generating pseudo-negative populations even when its intrinsic reporter signal is invisible (Figure 3F). Taken together, we conclude that oTVB-L is suitable for oEnvB-RVΔG-targeting to particular populations *in vivo*.

### Generation of the Optimized oEnvE/oTVE System

**Figure 4:**
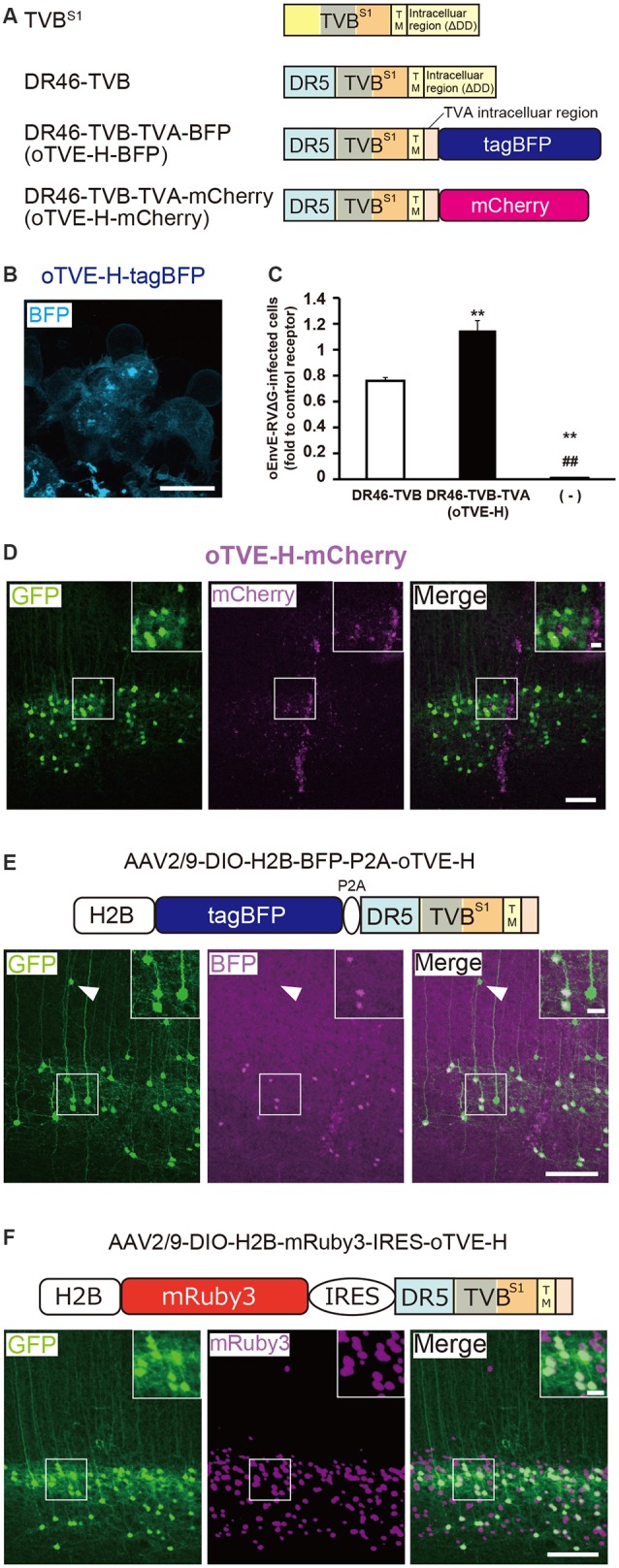
Generation of an optimized oEnvE/oTVE system for viral targeting. **(A)** Design of DR46-TVB variants fused to a red or blue fluorescent protein. DR46-TVB-TVA includes the extracellular-transmembrane region of DR46-TVB and the intracellular region of TVA^950^, according to the design of oTVB (see Figures 3A,B). **(B)** Membrane localization of DR46-TVB-TVA (oTVE-H)-BFP in HEK293t cells. The TagBFP signal was observed in the cell membrane. Signals were derived from native fluorescence. Scale bar: 20 μm. **(C)** Infection efficiency of oEnvE-RVΔG to cells expressing DR46-TVB or oTVE-H. Each column represents the mean ± SEM (*n* = 3). **P < 0.01 vs. DR46-TVB, ^##^*P* < 0.01 vs. oTVE-H (Tukey’s multiple tests). **(D)** Evaluation of *in vivo* function of oTVE-H-mCherry in Tlx3-Cre mice. Typical images of oEnvE-RVΔG-GFP-infected layer 5 neurons although the layer 5 neurons do not have visible expression of oTVE-H-mCherry. These data suggest that oTVE-H was sufficiently sensitive for oEnvE-RVΔG-GFP to infect layer 5 neurons expressing oTVE-H at a low expression level. Scale bar: 100 μm. The inset shows high magnifications of areas indicated by the white square. Signals were derived from native fluorescence. Scale bar: 20 μm. **(E)** The 2A-mediated oTVE-H expression system. oTVE-H was introduced by the H2B-tagBFP-P2A-oTVE-H cassette. oEnvE-RVΔG-GFP infected neurons expressing both nuclear H2B-tagBFP and oTVE-H in Tlx3-Cre mice. The arrowhead indicates non-specific infection to neurons in layers 2/3. Signals were derived from native fluorescence. Scale bar: 100 μm. The inset shows high magnifications of areas indicated by the white square. Scale bar: 20 μm. **(F)** The IRES-mediated the oTVE expression system for reliable detection of oTVE-H-expressing cells. oTVE-H was expressed downstream of the IRES sequence. oEnvE-RVΔG-GFP infected neurons expressing visible H2B-mRuby3 as well as oTVE-H in Tlx3-Cre mice. oEnvE-RVΔG-GFP-infected neurons expressed nuclear mRuby3. Signals were derived from native fluorescence. Scale bar: 100 μm. The inset shows high magnifications of areas indicated by the white square. Scale bar: 20 μm.

We next aimed to develop optimized DR46-TVB receptors that would allow specific targeting of EnvE viruses in the mammalian brain (Klucking and Young, 2004; Matsuyama et al., 2015). Intriguingly, DR46-TVB has been developed as an EnvE-specific receptor by replacing the TVB^S1^ CRD1 region with a corresponding region of death receptor 5 (Klucking and Young, 2004). Thus, we further optimized DR46-TVB as a tumor virus subgroup E (TVE)-specific viral receptor suitable for the rabies tracing experiments (Figure 4A). Interestingly, DR46-TVB shares the same transmembrane and cytoplasmic domains with the original TVB. As TVB did not show clear membrane localization in the mammalian cells, we endowed the clear membrane localization and the fluorescent-protein-tagging of DR46-TVB to efficiently and unambiguously visualize the starter neurons. For that purpose, we followed the same strategy as that used for oTVB. We swapped the intracellular domain of DR46-TVB with the intracellular domain of TVA to generate DR46-TVB-TVA and then we fused the C-terminus of the DR46-TVB-TVA to tagBFP or to mCherry to use it along with TVA^950E66T^-iRFP670 (Figure 4A, Supplementary Figure S2). We termed DR46-TVB-TVA “oTVE-H” (optimized DR46-TVB with a high affinity for oEnvE-pseudotyped viruses). Both oTVE-H-tagBFP and oTVE-H-mCherry were localized on the cell membrane of the transfected HEK293t cells (Figure 4B).

Next, we determined whether the cells expressing oTVE-H can be infected with oEnvE-RVΔG *in vitro* (Figure 4C). We transfected HEK293t cells with the oTVE-H-coding plasmid and subsequently applied oEnvE-RVΔG. oEnvE-RVΔG significantly infected the oTVE-H-expressing HEK293t cells. The infection efficiency of oTVE-H was higher than that of DR46-TVB. The higher infection efficiency of oTVE-H can be attributed to its higher membrane localization compared with that of DR46-TVB. We further investigated the specificity and cross-infectivity of oTVE-H to oEnvX-RVΔG *in vitro* (Table 2). We applied oEnvX-RVΔG to oTVE-H-expressing HEK cells and we found that oEnvE-RVΔG infected them, whereas oEnvA-RVΔG and oEnvB-RVΔG did not. Consequently, these results suggest that oTVE-H can be used as a specific receptor for oEnvE-RVΔG targeting.

To evaluate whether oTVE-H allows viral targeting to a specific population *in vivo*, we injected Cre-dependent AAV expressing oTVE-H-mCherry (AAV2/9-CAG-DIO-oTVE-H-mCherry) in the V1 of layer 5-specific Tlx3-Cre mice, as previously performed with oTVA-L and oTVB, and then injected oEnvE-RVΔG-GFP into the same location (Figure 4D). We observed some GFP-expressing neurons in layer 5 around the injection site, but not any mCherry-expressing neurons. No infection with oEnvE-RVΔG-GFP was observed in mice lacking oTVE-H expression (Supplementary Figure S1C). It is plausible that although a small amount of oTVE-H-mCherry was expressed in layer 5 neurons at the level of an invisible fluorescent reporter signal, its affinity to oEnvE-RVΔG was too high; thus, oTVE-H recognized oEnvE-RVΔG and led to viral infection.

As previously performed with oTVA-L or oTVB-L for unambiguous labeling of target cells *in vivo* (Figures 2, 3), we sought to reduce oTVE-H affinity. Previous reports have indicated that the CRD2 region of TVB^S1^ is important for ASLV-E infection, and the recognition site is composed of three residues in CRD2 (Klucking and Young, 2004). Thus, according to the TVB^S1^ mutation (Klucking and Young, 2004), we generated CRD region-deleted oTVE-H (DR46-TVB^ΔCRD2^-TVA and DR46-TVB^ΔCRD3^-TVA) and single mutated oTVE-H receptors (DR46-TVB^Y102A^-TVA, DR46-TVB^N107A^-TVA, and DR46-TVB^N108A^-TVA) fused to tagBFP (Supplementary Figure S3). The tagBFP signals of every receptor mutant were observed on the membrane of the transfected-HEK293t cells but none of them recognized oEnvE-RVΔG (data not shown).

Clear labeling of starter neurons is essential to determine the post-synaptic neurons in rabies tracing experiments, although the cells expressing oTVE-H-mCherry can be infected with oEnvE-RVΔG even when its expression is too low to be detected. It is conceivable that oTVE-H is inefficiently expressed in the mouse brain and its fused-reporter signal is barely detected. Thus, to reliably visualize target cells expressing oTVE-H, we first used a 2A-mediated bicistronic expression system (Szymczak et al., 2004; Chng et al., 2015) with a fluorescence reporter but without immunostaining (Figure 4E). We designed a bicistronic viral vector that expressed the two nuclear-localized transgenes tagBFP and oTVE-H (AAV2/9-CAG-DIO-H2B-tagBFP-p2A-oTVE-H). Then, we injected this AAV into the V1 of Tlx3-Cre mice and 2–3 weeks later oEnvE-RVΔG-GFP was injected into the same site. Seven days after RV injection, we counted the number of GFP/tagBFP-double-positive neurons in the V1. The results showed that 84.4 ± 4.4% of the GFP^+^ neurons also expressed H2B-tagBFP (total 547/659 neurons from three mice). GFP-positive and H2B-tagBFP-negative neurons were observed outside layer 5 of the cortex. This is because leak expression of oTVE-H derived from AAV-DIO vectors induced oEnvE-RVΔG-GFP infection. These results suggest that the 2A-system permits labeling and detection of the starter neurons with a fluorescence reporter and that only a small amount of oTVE-H is needed for the infection of oEnvE-RVΔG even at a barely visible expression level of its reporter fluorescence.

To reliably label and detect oTVE-H-expression cells for circuit mapping, we hypothesized that if oTVE-H expression could be reduced and its reporter fluorescence could be increased, we would be able to unambiguously detect oEnvE-RVΔG-infected neurons without immunostaining. Thus, we used another bicistronic system, the internal ribosome entry site (IRES) system, which can adjust oTVE-H expression to a lower level (Figure 4F). We generated AAV vectors that allow for lower expression of oTVE-H (AAV2/9-CAG-DIO-H2B-mRuby3-IRES-oTVE-H). This system enables bicistronic expression but the expression level of the second gene (oTVE-H) after the IRES sequence is lower than that of the first gene (H2B-mRuby3; Mizuguchi et al., 2000; Mardinly et al., 2018). We injected AAV2/9-CAG-DIO-H2B-mRuby3-IRES-oTVE-H into the V1 and then applied oEnvE-RVΔG-GFP into the same injection site (Figure 4F); 99.0 ± 0.4% of GFP^+^ cells also expressed H2B-mRuby3 (total 590/595 neurons from three mice). Compared to the 2A system that enables the bicistronic expression of two genes at the same expression level (Chng et al., 2015), the IRES-mediated oTVE expression system facilitates the unambiguous detection of oEnvE-RVΔG-infected neurons without the need of immunostaining signal enhancement (Figures 4E,F).

Next, we examined whether the oEnvE/oTVE system can be used for circuit tracing. We injected AAV expressing both oTVE and oG in a Cre-dependent manner into the mouse V1 of Tlx3-Cre mice. Three weeks after the AAV injection, oEnvE-RVΔG-GFP was injected into the same injection site. Ten days after the rabies injection, we found GFP^+^ presynaptic cells in the V1 and dorsal lateral geniculate nucleus (dLGN; Supplementary Figure S4), which is consistent with the previous report (Kim et al., 2015). These results suggest that the oEnvE/oTVE system could be used for trans-synaptic circuit tracing.

In the present study, we developed the chimeric envelope protein, oEnvE, and its cognate high-affinity receptor, oTVE. AAV vectors carrying the IRES-mediated bicistronic system could introduce oTVE-H in starter cells for oEnvE-RVΔG targeting. The oEnvE/oTVE system can be used for circuit mapping for particular cell populations in the mammalian nervous system.

### Independence Between the oEnvA/oTVA and oEnvB/oTVB Systems *in vivo*

**Figure 5:**
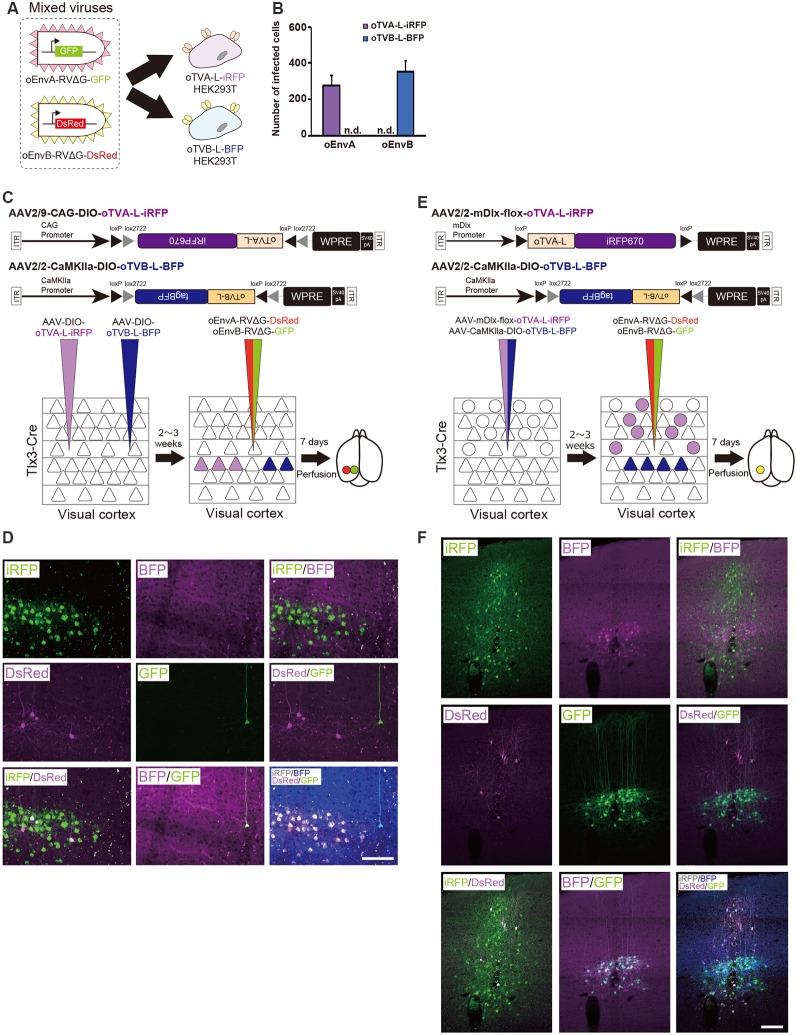
Independence of the oEnvA/oTVA and oEnvB/oTVB systems. **(A)** *In vitro* evaluation of specificity of oEnvA-RVΔG-GFP and oEnvB-RVΔG-DsRed to cells expressing either oTVA-L-iRFP or oTVB-L-BFP. oEnvA-RV and oEnvB-RV mixed viruses applied to HEK293t cells expressing oTVA-L-iRFP or oTVB-L-BFP transitorily. **(B)** No cross-infectivity of oEnvA-RVΔG-GFP and oEnvB-RVΔG-DsRed to cells expressing either oTVA-L-iRFP or oTVB-L-BFP. Evaluation of *in vitro* infectious specificity. Each column represents the mean ± SEM (*n* = 3). n.d.: not detected. **(C)** *In vivo* infectious specificity of oTVA-L-iRFP and oTVB-L-BFP that are expressed in two different locations of the brain. AAV-CAG-DIO-oTVA-L-iRFP and AAV-CaMKIIa-DIO-oTVB-L-BFP were injected in two different locations of Tlx3-Cre mice, 800 μm apart. A mixture of oEnvA-RVΔG-DsRed and oEnvB-RVΔG-GFP was injected between the two injection sites of AAV. **(D)** Non-co-infection with oEnvA-RVΔG and oEnvB-RVΔG. Signals were derived from native fluorescence. Scale bar: 100 μm. **(E)** *In vivo* infection specificity of oTVA-L-iRFP and oTVB-L-BFP that are expressed in two different cell types. oTVA-L-iRFP and oTVB-L-BFP are exclusively expressed by a distinct promotor and a transgenic Cre line. Inhibitory neurons were targeted by the inhibitory neuron-specific mDlx promoter and layer 5 neurons recombined the AAV genome to excise the flox cassette and eliminate gene expressions. Excitatory neurons in layer 5 of the cortex were targeted by a combination of the excitatory neuron-specific CaMKIIa promoter and the layer 5-specific Tlx3-Cre line. AAV-mDlx-oTVA-L-iRFP and AAV-CaMKIIa-DIO-oTVB-L-BFP were co-injected into the V1 of Tlx3-Cre mice. oEnvA-RVΔG-DsRed and oEnvB-RVΔG-GFP were co-injected into the location of the AAV injection. **(F)** No cross-infectivity between oEnvA-RVΔG and oEnvB-RVΔG. Signals were derived from native fluorescence. Scale bar: 100 μm.

To differentially dissect and visualize two independent cell populations with fluorescent proteins, we next applied both the oEnvA/oTVA and oEnvB/oTVB systems to the mouse brain. To trace circuits with RVΔG, we needed to reliably label starter cells and introduce two genes RV-G and oTVX in the starter cells. For that purpose, we used a 2A element to express both genes from the AAV genome. To label two different populations, we selected oTVA-L and oTVB-L, as both are low-affinity receptors to the corresponding envelopes (Figures 2E,F, 3I). Initially, to assess the specificity and cross-infectivity of oTVA-L-iRFP and oTVB-L-tagBFP, we performed an *in vitro* assay by preparing the oTVA-L-iRFP- and oTVB-L-tagBFP-expressing HEK293t cells individually and then applied a mixture of oEnvA-RVΔG-GFP and oEnvB-RVΔG-DsRed into each culture (Figure 5A). Three days after the viral infection, 100% of the GFP^+^ cells expressed iRFP670 and 0% of the GFP^+^ cells expressed BFP (Figure 5B). Similarly, 100% of the DsRed^+^ cells expressed BFP and 0% of the DsRed^+^ cells expressed iRFP670. These results indicate that oTVA-L and oTVB-L, respectively recognized oEnvA-RVΔG and oEnvB-RVΔG without cross-reaction.

Next, we investigated the *in vivo* specificity and cross-infectivity of oEnvA-RVΔG and oEnvB-RVΔG. We introduced oTVA-L and oTVB-L separately in two different locations of the mouse brain (Figure 5C). We injected oTVA-L-iRFP-expressing AAV and oTVB-L-tagBFP-expressing AAV at the medial and lateral V1 (0.8 mm apart) of Tlx3-Cre mice, respectively, and subsequently injected a mixture of oEnvA-RVΔG-DsRed and oEnvB-RVΔG-GFP between the AAV-injection sites. Seven days after the RV injection, we sacrificed the mice and observed the injection sites of the sectioned brain. Indeed, iRFP^+^ and tagBFP^+^ cells were exclusively distributed in the V1 (from three mice). No co-infection was observed between the oTVA-L-iRFP- and oTVB-L-tagBFP-expressing AAV (Figure 5D). All oEnvA-RVΔG-derived DsRed^+^ neurons also expressed iRFP670. Moreover, most oEnvB-RVΔG-derived GFP^+^ neurons also expressed BFP; the rest of the GFP^+^ neurons there was no BFP signal observed, but all GFP^+^ neurons were observed in the medial V1 injection site of the oTVB-L-tagBFP-expressing virus (Supplementary Table S1). Thus, the receptors did not recognize the non-corresponding envelopes.

To further demonstrate the specificity and independence of the oEnvA/oTVA and oEnvB/oTVB systems, we constructed two helper viruses: the first one carried an inhibitory-neuron-specific mDlx promotor (Dimidschstein et al., 2016) that allowed the inhibitory neurons to express oTVA-L-iRFP, and the other expressed oTVB-L-tagBFP in Tlx3-Cre mice, a layer 5 excitatory neuron-specific line, in a Cre-dependent manner (Figure 5E). We injected the mixture of these two AAVs to the V1 of Tlx3-Cre mice and then the mixture of oEnvA-RVΔG-DsRed and oEnvB-RVΔG-GFP to the location of the AAV injection. Most DsRed-expressing neurons were iRFP-positive while majority of GFP-expressing neurons were tagBFP-positive (Figure 5F, Supplementary Table S1). No GFP/DsRed-double-positive cells were observed in the injected mice. These results indicate that the oEnvA/oTVA-L and oEnvB/oTVB-L systems targeted inhibitory and layer 5 excitatory neurons in the mouse cortex, respectively, in a mutually exclusive manner.

In conclusion, we demonstrated that the oEnvA/oTVA-L and oEnvB/oTVB-L systems can be used in simultaneous *in vivo* targeting of RVΔG in single animals. Using the oEnvA/oTVA-L and oEnvB/oTVB-L systems for RVΔG enables differentially dissecting and visualizing two independent cell populations with fluorescent proteins.

### Simultaneous Trans-synaptic Tracing Using the oEnvA/oTVA and oEnvB/oTVB Systems

To perform a simple proof-of-concept experiment for simultaneous multiplex circuit tracing, we aimed to dissect multiple neural circuits using the oEnvA/oTVA and oEnvB/oTVB systems that we developed for the purposes of the present study. V1 neurons are heterogeneous; excitatory neurons even on the same layer have different connection patterns, and inhibitory neurons have different biochemical markers, morphological features, electrophysiological properties, and connection patterns. Layer 5 neurons in the V1 comprise at least two different cell types depending on their projection targets (Brown and Hestrin, 2009; Lur et al., 2016). The first one projects to the cortical areas, such as the higher visual areas, and the other to subcortical areas, such as the superior colliculus (SC; Brown and Hestrin, 2009; Lur et al., 2016). These two types of layer 5 neurons that project to different target regions are locally intermingled.

**Figure 6:**
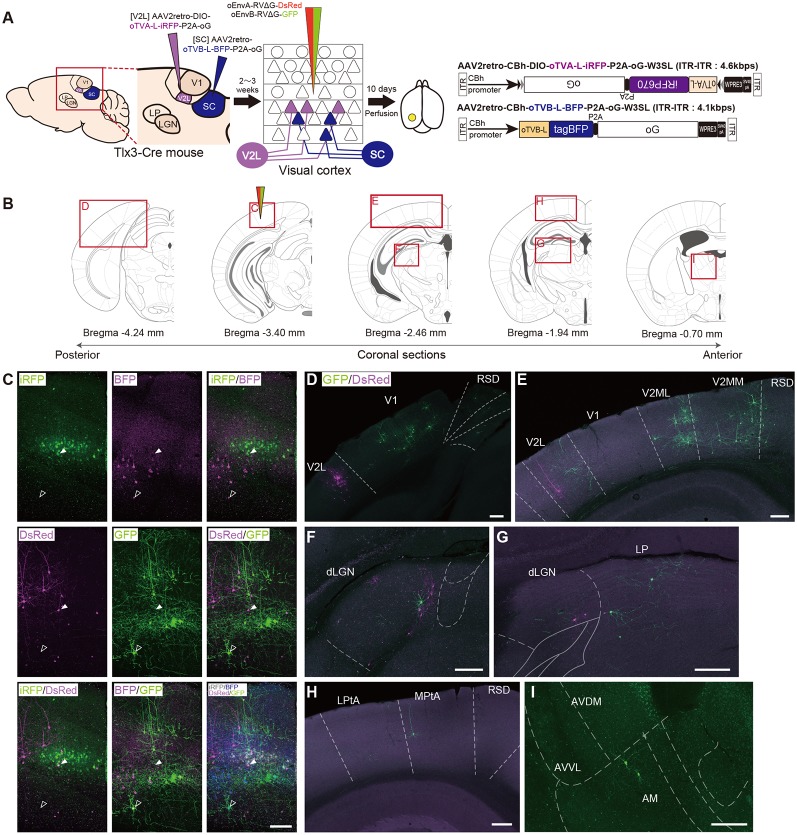
Multiplex circuit tracing with the oEnvA/oTVA and oEnvB/oTVB systems. **(A)** Multiplex monosynaptic tracing of two distinct classes of layer 5 neurons targeting either other cortical or subcortical areas. Retrograde AAVs were injected into two different locations of layer 5-specifc Tlx3-Cre mice: AAV2retro-CBh-DIO-oTVA-L-iRFP-P2A-oG and AAV2retro-CBh-oTVB-L-BFP-P2A-oG in the lateral area of the higher visual cortex (V2L) and superior colliculus (SC), respectively. V2L-projecting V1 neurons in layer 5 expressed both oTVA-L-iRFP and oG while SC-projecting V1 neurons expressed both oTVB-L-BFP and oG. After the AAV injections, oEnvA-RVΔG-DsRed and oEnvB-RVΔG-GFP were co-injected into the V1. **(B)** Coronal brain sections illustrating anatomical regions shown in (**C**–**I**; adapted from The Mouse Brain in Stereotaxic Coordinates, 3rd edition, Paxinos and Franklin, 2008; **G**). **(C)** Viral targeting to two distinct classes of layer 5 neurons depending on projection targets. oTVA-L-iRFP-expressing starter neurons were specifically infected with oEnvA-RVΔG-DsRed, whereas oTVB-L-BFP-expressing starter neurons were infected with oEnvB-RVΔG-GFP. DsRed and GFP signals were enhanced by immunostaining. Closed arrowheads indicate oTVA-L-iRFP^+^/oEnvA- RVΔG-DsRed^ +^ starter neurons. Opened arrowheads indicate oTVB-L-BFP^+^/oEnvB-RVΔG-GFP^+^ starter neurons. Scale bar: 100 μm. **(D–I)** Distributions of presynaptic neurons connected to two distinct classes of layer 5 neurons. Neurons trans-synaptically infected with RVΔG-DsRed and RVΔG-GFP were distributed in the V1, V2L, and RSD **(D)**, V1, V2L, V2ML, V2MM, and RSD **(E)**, dLGN **(F)**, dLGN and LP **(G)**, LPtA, MPtA, and RSD **(H)**, AM, AVDM, and AVVL **(I)**. DsRed and GFP signals were enhanced by immunostaining. Scale bar: 200 μm. Abbreviations: AM, anteromedial thalamic nucleus; AVDM, anterovent thalamic nucleus, dorsomedial part; AVVL, anteroventral thalamic nucleus, ventrolateral part; RSD, retrosplenial dysgranular cortex; dLGN, dorsal lateral geniculate nucleus; LP, lateral parietal association cortex; LPtA, medial parietal association cortex; MPtA, lateral posterior thalamic nucleus; V2L, secondary visual cortex, lateral area; V2ML, secondary visual cortex, mediolateral area; V2MM, secondary visual cortex, mediomedial area.

To distinguish these two different layer-5 cell types and their corresponding presynaptic networks, we introduced the oEnvA/oTVA and oEnvB/oTVB systems to each population (Figure 6A). The AAV2retro capsid allows AAV to retrogradely infect projection neurons through axon terminals (Tervo et al., 2016). We first planned to develop an AAV2retro-CAG-oTVA-L-iRFP-P2A-oG (ITR-ITR: >4.9 kbps) that would simultaneously express both oTVA-L-iRFP and the optimized rabies glycoprotein oG. However, the AAV genome is limited (<4.7 kbps; Dong et al., 1996; Allocca et al., 2008), so we could not obtain sufficient AAV to be usable *in vivo*. Then, to maximize space for transgenes within the AAV packaging capacity without compromising transgene expression, we used a shorter universal promotor and a shorter polyA sequence to accommodate TVX, oG, and a fluorescent reporter such as, pAAV-CBh-oTVA-L-iRFP-P2A-oG-WPRE3-SV40 late polyA (ITR-ITR: approximately 4.3 kbps; Choi et al., 2014).

We administered two injections of retrograde AAVs to Tlx3-Cre mice. First, Cre- and projection-dependent, retrograde AAV expressing oTVA-L-iRFP and oG (AAV2retro-CBh-DIO-oTVA-L-iRFP-P2A-oG-WPRE3-SV40 late polyA) were injected into the lateral higher visual area (V2L: the putative area LM) to introduce both TVA and oG to V2L-projecting layer 5 neurons (Kim et al., 2016; Figures 6A,B). Second, projection-dependent, retrograde AAV expressing oTVB-L-tagBFP and oG (AAV2retro-CBh-oTVB-L-BFP-P2A-oG-WPRE3-SV40 late polyA) was injected in the SC to introduce both oTVB and oG to SC-projecting neurons (Lur et al., 2016; Figures 6A,B). iRFP^+^ cells were observed in layer 5 of the mouse V1, suggesting that V2L-projecting layer 5 neurons of V1 were labeled in a Cre-dependent and retrograde manner (Figure 6C). BFP^+^ neurons were also observed in layer 5, suggesting that the SC-projecting V1 neurons were retrogradely labeled. Using the conditional AAVs in Tlx3-Cre mice, we succeeded in specific labeling of layer 5 neurons projecting to either the V2L or SC area with oTVA-L or oTVB-L, respectively.

To identify their presynaptic inputs, we next injected oEnvA-RVΔG-DsRed and oEnvB-RVΔG-GFP into the V1 of the AAV-injected Tlx3-Cre mice 2–3 weeks after the AAV injections. Ten days after the rabies viral injection, we sacrificed the animals for histological analysis. Most DsRed^+^ cells in upper layer 5 were positive for iRFP670, indicating that oEnvA-RVΔG-DsRed infected oTVA-L-iRFP^+^ cells in upper layer 5 of the V1 and that the DsRed/iRFP-double-positive cells were starter cells for trans-synaptic tracing of RVΔG-DsRed. RVΔG-DsRed spread to the presynaptic cells following trans-complementation with oG (Figures 6B–I). DsRed-positive and iRFP670-negative cells were mainly observed in layer 2/3 and 4 of the V1 (Figure 6C), V2L (Figures 6D,E), and dLGN (Figures 6F,H). Likewise, most GFP^+^ cells in lower layer 5 were also tagBFP-positive, indicating that oEnvB-RVΔG-GFP infected the oTVB-L-tagBFP^+^ cells in lower layer 5 of the V1, while the GFP/BFP-double-positive cells were the starter cells for trans-synaptic tracing of RVΔG-GFP. The GFP^+^ cells were more widely spread in V1, the higher visual areas V2ML and V2MM (Figure 6E), association cortical areas (medial parietal association; Figure 6G), and various thalamic areas including the dLGN, LP (Figures 6F,H), and anterior thalamic nuclei (Figure 6I). Additionally, GFP^+^ and DsRed^+^ neurons were not observed in any of the brain regions. These results suggest that V2L-projecting V1 neurons on layer 5 received inputs from V1 local circuits, feedback from higher visual areas, and visual-related thalamic areas, whereas SC-projecting V1 neurons received inputs from V1 local circuits, feedback from higher visual and association cortical areas, and visual and non-visual thalamic nuclei.

Therefore, we conclude that the multiplexed neural circuit tracing with RVΔG enabled the simultaneous dissection of multiple neural circuits in the brain.

### Labeling of Common Input to Different Cell Populations With Multiplex Circuit Tracing

In the next set of experiments, we examined whether common input to two distinct cell populations can be detected by multiplex circuit tracing *in vivo*, which can be detected as co-infected cells with two viral vectors in multiplex circuit tracing. Many lines of evidence have shown that dLGN neurons send projections to neurons in layers 4, 5, and 6 of the V1 (Blasdel and Lund, 1983; Antonini et al., 1999; Constantinople and Bruno, 2013; Kim et al., 2015). A recent study revealed that parvalbumin (PV)-expressing inhibitory neurons in the V1 receives input from dLGN neurons (Kim et al., 2016).

**Figure 7:**
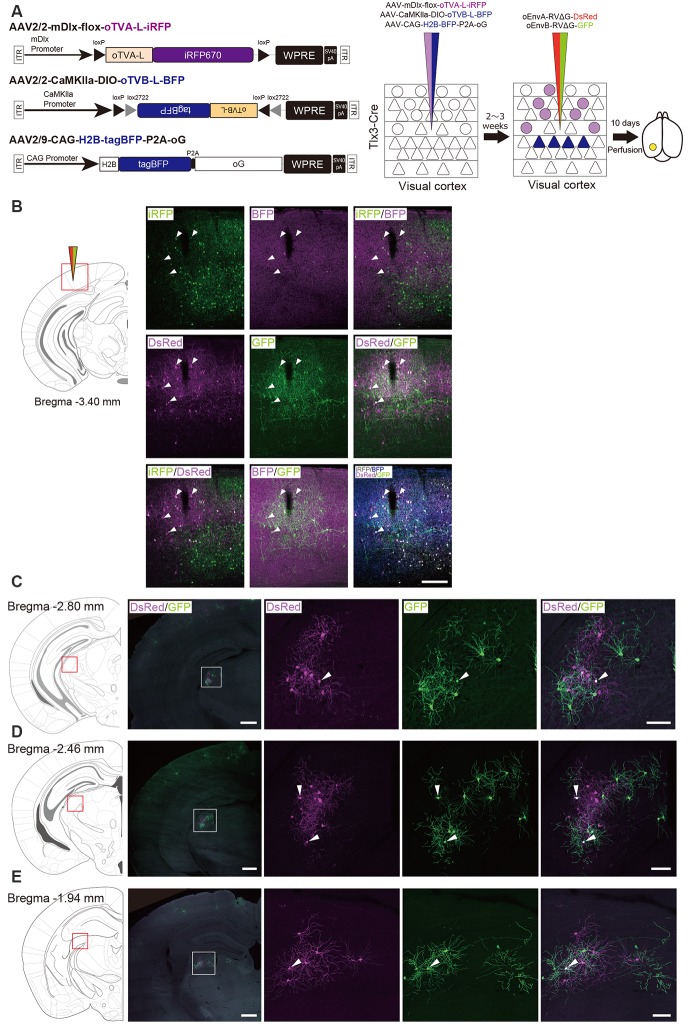
Labeling of common input with multiplex circuit tracing. **(A)** Multiplex monosynaptic tracing of two distinct classes of layer 5 excitatory or inhibitory neurons. Mixed AAVs were injected into the V1 of layer 5-specifc Tlx3-Cre mice: AAV2/2-mDlx-flox-oTVA-L-iRFP, AAV2/2-CaMKIIa-DIO-oTVB-L-BFP, and AAV2/9-CAG-H2B-tagBFP-P2A-oG. After the AAV injection, oEnvA-RVΔG-DsRed and oEnvB-RVΔG-GFP were co-injected into the same location of the V1. **(B)** Viral targeting to two distinct classes of V1 neurons depending on cell types. oTVA-L-iRFP-expressing inhibitory neurons were specifically infected with oEnvA-RVΔG-DsRed, whereas oTVB-L-BFP-expressing excitatory neurons in layer 5 were infected with oEnvB-RVΔG-GFP. The rabies glycoprotein oG was expressed in cells positive for nuclear tagBFP. Closed arrowheads indicate presynaptic neurons co-infected with both oEnvA-RVΔG-DsRed and oEnvB-RVΔG-GFP. DsRed and GFP signals were enhanced by immunostaining. Scale bar: 200 μm. **(C–E)** Low and high magnification images of dLGN neurons monosynaptically connected to two distinct classes of V1 neurons. DsRed and GFP signals were enhanced by immunostaining. Closed arrowheads indicate presynaptic neurons co-infected with both oEnvA-RVΔG-DsRed and oEnvB-RVΔG-GFP. Scale bar of overview: 500 μm. Scale bar of dLGN view: 100 μm. Coronal brain sections illustrating anatomical regions shown in (**B–E**; adapted from The Mouse Brain in Stereotaxic Coordinates, 3rd edition, Paxinos and Franklin, 2008).

Based on these reports, we performed multiplex RV tracing from the inhibitory neurons and layer 5 neurons of the V1 in single animals to determine whether inhibitory neurons and layer 5 excitatory neurons of the V1 share common input (Figure 7A). Cortico-coritcal layer 5 neurons of the V1 were labeled with Cre-dependent AAV expressing oTVB under the excitatory-neuron-specific CaMKIIa promoter in layer 5-specific Tlx3-Cre mice. Inhibitory neurons of the V1 were labeled with AAV expressing oTVA under the inhibitory-neuron-specific mDlx promoter using the flox cassette that is excised by recombination in the Cre-expressing cells to eliminate oTVA expression in layer 5 excitatory neurons of Tlx3-Cre mice. Using this viral system, oTVA and oTVB can be exclusively introduced to layer 5 excitatory neurons and inhibitory neurons, respectively. Additionally, to complement RVΔG *in trans*, we co-injected AAV expressing the rabies glycoprotein oG under the strong ubiquitous CAG promotor, as the level of gene expressions driven by the CaMKIIa and mDlx promotors differs. Three weeks after the AAV injection, oEnvA-RVΔG-DsRed and oEnvB-RVΔG-GFP were co-injected in the same location of the V1 injection of AAVs. We observed oTVA-L-iRFP-positive cells in inhibitory neurons across layers and oTVB-L-BFP-positive cells (cell membrane-localized BFP) in layer 5 of the V1. We also found GFP^+^ and DsRed^+^ cells, indicating that common input was detected as co-infection with both oEnvA-RVΔG-DsRed and oEnvB-RVΔG-GFP. Interestingly, GFP^+^/DsRed^+^ neurons were distributed in the V1 (Figure 7B) and dLGN (Figures 7C–E). These results indicate that inhibitory neurons and layer 5 excitatory neurons of the V1 share common input from V1 local circuits and from dLGN neurons.

We found that dLGN neurons provide divergent input to both layer 5 excitatory and inhibitory neurons of the V1 in the mouse visual system. The common input to both layer 5 excitatory and inhibitory neurons could provide a sub-network structure crucial for shaping the receptive field properties of V1 neurons. Taking together, we conclude that multiplex neural circuit tracing with RVΔG allows detecting common input to different cell populations and analyzing complex computations between circuits.

## Discussion

### Multiplex Neural Circuit Tracing With G-Deleted Rabies Viral Vectors

The brain is a complex and dynamic structure composed of heterogeneous neuronal populations that are organized in distinct neural circuits to generate perception and behavior (Harris and Mrsic-Flogel, 2013; Tasic et al., 2016, 2018). Information is processed not only through paralleled, independent circuits but also by integration between circuits in the nervous system including the retina and brain (Nassi and Callaway, 2009). Much progress has been made toward elucidating cell-type-specific connectivity at the whole-brain level. However, information integration in the brain remains elusive. Thus, dissection of the interaction between intermixing circuits will provide better opportunities to reveal the neural circuits and computations underlying integration of information processing. Here, we multiplexed the infection systems of RVΔG and demonstrated their implementation in the mouse brain. We introduced oEnvA/oTVA, oEnvB/oTVB, and oEnvE/oTVE systems to simultaneously target distinct cell populations with RVΔG. Our work provided proof-of-concept for multiplex circuit tracing with RVΔG in a single animal.

Our *in vivo* proof-of-concept experiment showed the simultaneous labeling of the two distinct classes of layer 5 neurons targeting either other cortical or subcortical areas. Using both the oEnvA/oTVA and oEnvB/oTVB systems, we differentially labeled two distinct populations with different colors in single animals. Their presynaptic inputs were also labeled with trans-synaptic spread of different-colored RVΔG. We found that SC-projecting layer 5 neurons of the V1 received input from local circuits and motor-related areas, while LM-projecting layer 5 neurons of the V1 received input from local circuits and visual-related areas including higher visual areas. We did not find any connections between SC-projecting layer 5 neurons and LM-projecting layer 5 neurons in V1 as co-infection. This result is consistent with the recent study by Maruoka et al. (2017). Maruoka et al. (2017) revealed lattice organization composed of cell type-specific microcolumns in layer 5; subcortical projection neurons formed radial clusters called microcolumns, while cortical projection neurons were also organized into microcolumns that were aligned radially in an orientation parallel to the microcolumns composed of subcortical projection neurons. Maruoka et al. (2017) also demonstrated that subcortical projection neurons were rarely connected with cortical projection neurons whereas subcortical projection neurons had reciprocal connections with each other and cortical projection neurons were also interconnected. In addition, we did not observe any co-infected cells with two rabies viral vectors outside V1, suggesting that the presynaptic network organization of layer 5 neurons differs depending on the output target; layer 5 neurons in V1 pool distinct information depending on their projection target. However, it is difficult to conclude that there is no common input between SC-projecting and LM-projecting layer 5 neurons in the V1 in this set of experiments. Although the SC-projecting layer 5 starter neurons were distributed in the vicinity of the LM-projecting starter neurons in the V1, the retinotopic location of SC-projecting layer 5 starter cells can be mismatched with that of LM-projecting layer 5 starter cells. It is possible that the cells responsible for the same location of the visual space can interact with each other and their presynaptic networks may overlap, whereas the cells responsible for different locations of the visual space may not share common input.

In another set of experiments, the multiplex circuit tracing labeled the common input to different cell populations; we found that dLGN neurons were directly connected to both cortico-cortical layer 5 neurons and inhibitory neurons of the mouse V1. It is known that the most canonical thalamocortical connection is between dLGN neurons and layer 4 neurons of the V1. However, Kim et al. (2016) demonstrated using rabies tracing that PV-expressing inhibitory neurons in the V1 receive input from dLGN neurons. Additionally, they revealed that layer 5 excitatory neurons in the V1 also receive input from dLGN neurons (Kim et al., 2015). Several lines of evidence have indicated that dLGN neurons provide collateral connections to layer 5 and 6 neurons of the V1, in addition to layer 4 neurons (Blasdel and Lund, 1983; Antonini et al., 1999). To our knowledge, no study has revealed that layer 5 and inhibitory neurons of the V1 share common excitatory input from dLGN neurons. This divergent thalamocortical input could play unique roles in shaping the receptive field properties of V1 neurons. Further physiological studies are warranted to determine how the common input participates in circuit computation and visual information processing in the V1.

### Viral Receptors With Higher or Lower Affinity

TVA fused to a fluorescent protein has been used to visualize TVA-expressing starter cells: TVA^950^-EYFP (Faget et al., 2016), TVA^950^-mCherry (Watabe-Uchida et al., 2012), and the low-affinity mutant TVA^950E66T^-mCherry (Miyamichi et al., 2013). TVA^950^-EYFP and TVA^950^-mCherry are highly sensitive to viral infection despite low expression of TVA^950^ caused by leak expression even though their fluorescent reporters (EYFP and mCherry) fused to TVA^950^ were invisible. For example, in transgenic mice expressing Cre in a specific cell population, Cre-negative cells can express a small amount of TVA^950^ due to leak expression, which leads to non-specific infection with EnvA-RVΔG. However, when using TVA^950E66T^-mCherry for rabies viral targeting in Cre-expressing transgenic mice, oEnvA-RVΔG cannot infect the cells expressing a small amount of TVA^950E66T^-mCherry, which are invisible under a fluorescent microscope because of the low affinity of TVA^950E66T^ to oEnvA (Rong et al., 1998; Miyamichi et al., 2013). The strength of this TVA^950E66T^-mCherry constitutes clear and reliable visualization of starter neurons in rabies tracing experiments because high expression of TVA^950E66T^-mCherry is required for oEnvA-RVΔG infection. Thus, a lower-affinity version of TVA (oTVA-L) is ideal for rabies viral tracing.

oEnvA-RVΔG, oEnvB-RVΔG, and oEnvE-RVΔG showed specific infectivity to cells expressing their corresponding receptors, TVA^800^, TVB^S3^, and DR46-TVB (Figure 1C and Table 1). Based on this specificity, we developed both higher-affinity and lower-affinity receptors, termed oTVB-H, oTVB-L, and oTVE-H. oTVB-H and oTVB-L are the respective higher and lower-affinity versions of TVB. oTVE-H is a receptor with higher affinity to oEnvE-RVΔG. Here, we propose to use optimal viral receptors depending on the purpose and design of experiments.

For cell-type-specific circuit tracing, using transgenic mouse lines expressing Cre, Flp, or tTA in a specific population is the most reliable and straightforward approach to define starter cells in mice. For that purpose, viral receptors with lower affinity, such as oTVA-L and oTVB-L, are recommended because leak expressions of viral receptors can be ignored. Typically, we used AAV vectors for introducing oTVA-L and oTVB-L and fluorescent reporters. In this study, we also developed high-affinity receptors, oTVB-H and oTVE-H. Higher-affinity versions are also applicable for AAV-mediated circuit tracing along with the IRES system for introducing both receptors and fluorescent reporters as shown in Figure 4E. Notably, as TVA^800^, high-affinity type TVA (Marshel et al., 2010; Gray et al., 2011; Rancz et al., 2011; Wertz et al., 2015), was used for labeling the single cell network *in vivo*; both oTVB-H and oTVE-H are suitable for single-cell electroporation followed by single-cell-initiated RVΔG tracing.

### Application of Multiplex Circuit Tracing

**Figure 8:**
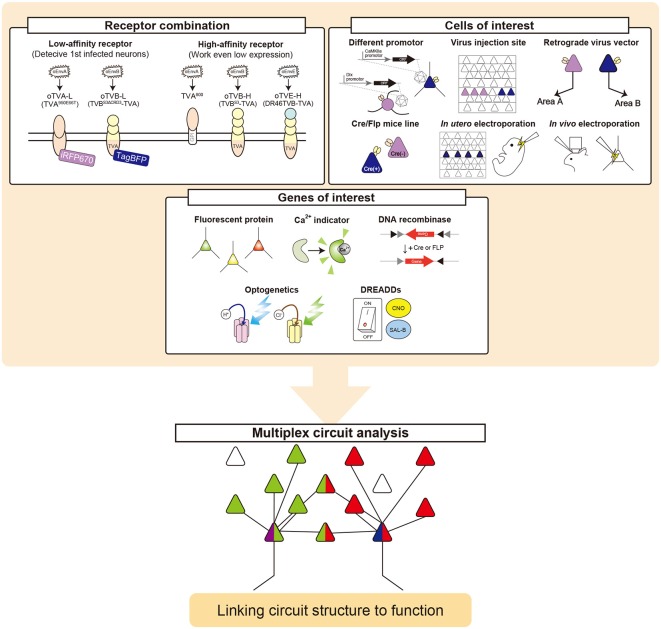
Multiplex circuit analysis with rabies viral vectors. Simultaneous RV trans-synaptic tracing can be achieved by designing three steps. (1) Select viral envelopes and their receptors. oEnvX/oTVX systems developed in the present study can be used for rabies viral targeting. (2) Select cells of interest. Cell-type-specific promoters, connection-mediated gene transfer, electroporation, and transgenic animals are available to target particular populations. (3) Select genes of interest. Fluorescent proteins, imaging probes, optogenetic/chemogenetic tools, and recombinases can be expressed from the rabies viral vectors.

Using the multiplex RVΔG tracing system that we developed in this study, various questions can be addressed regarding the circuit-level mechanisms of information processing. To utilize multiplex circuit tracing, we need to consider two parameters (Figure 8): (1) how to introduce the viral receptors (Table 3); and (2) which genes to express. (1) Using transgenic mouse lines marking specific cell populations is the most straightforward and easiest method to introduce both viral receptors and RV-G. Tissue-specific promoters (e.g., human synapsin promoter; pan-neurons), brain-region-specific promoters (e.g., Ple67/FEV promoter; Raphe nuclei neurons, Ple155/PCP2 promoter; cerebellum Purkinje cells; de Leeuw et al., 2016), cell-type-specific promoters (e.g., CaMKlla promoter; excitatory neurons, Dlx promoter; inhibitory neurons; Yaguchi et al., 2013; Dimidschstein et al., 2016), or activity-dependent promoters (e.g., E-SARE promoter; Kawashima et al., 2009, 2013) are also powerful when used within viral vectors. In conjunction with the transgenic Cre/Flp mouse lines or Cre/Flp-expressing viral vectors, Cre/Flp-dependent viral vectors carry the Flex or DIO/DO cassette that restricts expression by recombination (Gradinaru et al., 2010; Saunders et al., 2012; Fenno et al., 2014). tTA/rtTA-expressing transgenic mouse lines can be interfaced with AAV or HIV vectors carrying the tetO promoter. When retrograde infective vectors (e.g., AAV2retro, RV-G-pseudotyped lentivirus, Canine adenovirus type 2) are used, local area neurons can be separated by their projection area. An intersectional strategy using a combination of recombinases and activators can increase the specificity of target cells. (2) RVΔG can be used for both anatomical and physiological studies (Osakada et al., 2011; Wertz et al., 2015; Tian et al., 2016). RVΔG can accommodate a larger size of transgenes in their viral genome than can AAVs. Further, multiple genes can be expressed without using IRES or 2A elements (Osakada et al., 2011; Osakada and Callaway, 2013). Genetically-encoded sensors for calcium, voltage, and neurotransmitters are powerful to characterize connectionally-defined circuits (Chen et al., 2013; Marvin et al., 2013, 2019; Dana et al., 2016; Jing et al., 2018; Patriarchi et al., 2018; Sun et al., 2018; Abdelfattah et al., 2019; Inoue et al., 2019). Chemogenetic manipulation using rabies-mediated DREADD expressions (e.g., hm3dq, hm4di, PSAM, and KORD) can relate neuronal connectivity and activity to behavior (Armbruster et al., 2007; Alexander et al., 2009; Magnus et al., 2011; Vardy et al., 2015). Combining optogenetic tools (e.g., Channelrhodpsin2, ChRmine), the manipulation of connected populations will be feasible with implantation of optic fibers in the brain (Osakada et al., 2011; Tian et al., 2016) and even with two-photon excitation with single-cell resolution (Mardinly et al., 2018; Carrillo-Reid et al., 2019; Marshel et al., 2019).

**Table 3:** oEnvX-RVΔG receptors which are available.

Chimeric Envelope	Receptor Name	Fused Reporter	Structure	Detectivity of Starter Neurons	Sensibility	References
oEnvA	TVA^800^	−	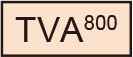	−	+++	Marshel et al. (2010)
	TVA^950^	mCherry EYFP	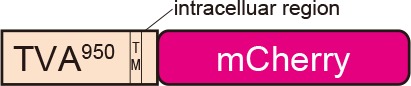	△	++	Watabe-Uchida et al. (2012) and Faget et al. (2016)
	TVA^950E66T^ (oTVA-L)	mCherry EYFP	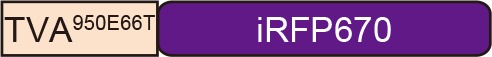	◎	+	Miyamichi et al. (2013) This study
oEnvB	TVB^S3^	−	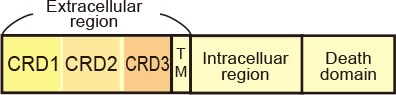	−	+	Brojatsch et al. (1996)
	TVB^S3^-TVA (oTVB-H)	tagBFP	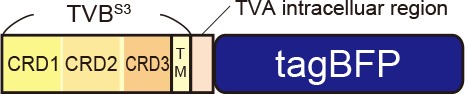	△	+++	This study
	TVB^S3ΔCRD3^-TVA (oTVB-L)	tagBFP	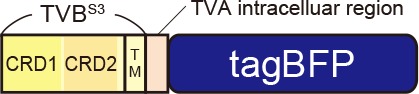	◯	++	This study
oEnvE	DR46-TVB	−	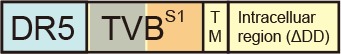	−	++	Klucking and Young (2004)
	DR46-TVB-TVA (oTVE-H)	mCherry tagBFP	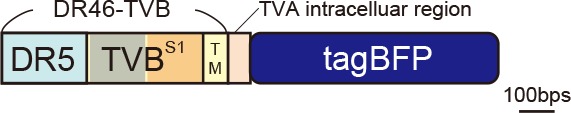
	△ (with IRES : ◎)	+++	This study

*In the detectivity of starter neurons, each symbol indicates the following: ◎: the fluorescence reporter detects starter neurons almost perfectly at >90%. ◯: the fluorescence reporter detects starter neurons at >80% and produces some pseudo-negative cells. △: the fluorescence reporter cannot easily detect starter neurons and produces many pseudo-negative cells*.

### Limitations and Future Challenges

Optical techniques have become indispensable for neuroscientific research in recent decades (Yang and Yuste, 2017). Genetically-encoded indicators of calcium, voltage, neurotransmitters, and metabolism as well as optogenetic and chemogenetic actuators allow multi-modal interrogation of neural circuit function (Luo et al., 2018). These multi-colored, genetically-encoded biosensors and actuators will fit our multiplex rabies viral tracing. However, the number of colors we can use is limited due to the limitation of genetically-encoded fluorescent proteins. Accordingly, the number of circuits we can simultaneously analyze is also currently limited.

To overcome these limitations, chemical tags could be a viable alternative to fluorescent proteins. Genetically-encoded chemical tags, such as Halo-tag, SNAP-tag, CLIP-tag, and TMP-tag, were labeled with synthetic fluorophores (Gautier et al., 2008; Los et al., 2008; Chen et al., 2012; Kohl et al., 2014). Interfacing chemical tagging with rabies tracing is also powerful for multiplexed circuit analyses because more choices of fluorescence spectrum and photochemical properties are available with chemical tags than with fluorescent proteins. Synthetic fluorophores cover the spectrum from the UV to near-IR range, expanding the possible spectrum range and the repertoire of fluorescence labels. Thus, chemical tagging will enable advances in multiplexed capacity and the applications of neural circuit tracing.

Rabies viral vectors are powerful tools for labeling synaptically-connected neurons throughout the brain. Despite the strength in anatomical studies, application of RVΔG tracing in physiological and behavioral studies remains limited because of its toxicity (Osakada et al., 2011; Wertz et al., 2015; Tian et al., 2016), although the toxicity of RVΔG viral vectors is much lower than that of other replication-competent viruses such as HSV. The viral toxicity derives from the trans-synaptic spread feature of replication-competent rabies vectors. It is notable that viral toxicity and trans-synaptic spread are in tradeoff. Several approaches have been developed to reduce the toxicity of RVΔG vectors. Large protein (L) deleted rabies viral vectors expressing Cre recombinase minimized the toxicity in transgenic mice expressing a reporter in a Cre-dependent manner (Chatterjee et al., 2018). This is because L-deleted RVΔG vectors are deficient in the replication of the RV genome. However, L-deleted RVΔG vectors do not replicate in infected neurons, thereby failing in viral spread to their presynaptic neurons due to lack of replication ability. Although trans-complementation of L and G allows replication of L-deleted RVΔG vectors and viral spread from starter cells, L-deleted RVΔG vectors will kill the L-expressing starter cells. Self-inactivating RVΔG also controlled its viral replication using PEST and TEV signals to extend the survival periods of infected neurons (Ciabatti et al., 2017). However, numerous researchers failed to reproduce the original report by Ciabatti et al. (2017), probably because rabies viruses obtain new mutations in the viral genome (Matsuyama et al., 2019). Further improvements of rabies viral vectors that have lower toxicity but maintain trans-synaptic spread ability are required for neural circuit research.

Revealing parallel processing through independent circuits and integrated processing through interaction between circuits is pivotal in understanding how the brain functions. The multiplex RV tracing that we developed in the present study will facilitate the in-depth understanding of the principles of neural circuits and computations in the brain.

## Data Availability Statement

All datasets generated for this study are included in the article/Supplementary Material.

## Ethics Statement

The animal study was reviewed and approved by the Animal Care and Use Committee in Nagoya University.

## Author Contributions

TS performed experiments. TS, NM, and FO analyzed the data. TS, NM, AA, and FO wrote the manuscript. FO designed the study and supervised this study.

## Conflict of Interest

The authors declare that the research was conducted in the absence of any commercial or financial relationships that could be construed as a potential conflict of interest.

## Footnotes

^1^https://www.r-project.org/

## Supplementary Material

FIGURE S1 Control injection of oEnvX-RVΔG in the absence of oTVX. No cells were infected with oEnvA-RVΔG-DsRed **(A)**, oEnvB-RVΔG-DsRed **(B)**, or oEnvE-RVΔG-GFP **(C)** without injection of AAV expressing corresponding receptor oTVX. These results indicate no sign of contamination by unpseudotyped RVΔG in oEnvA-RVΔG-DsRed **(A)**, oEnvB-RVΔG-DsRed **(B)**, and oEnvE-RVΔG-GFP **(C)**. Arrowheads indicate viral injection sites. Scale bar: 100 μm.

FIGURE S2 Protein sequence alignment for TVB^S3^, oTVB-H, and oTVB-L. Colored regions indicate functional domains: CRD1, CRD2, CRD3, and a transmembrane region. Gray boxes indicate absent or different amino acid.

FIGURE S3 Protein sequence alignment for DR46-TVB and oTVE-H. Colored regions indicate the functional domains: death receptor 5, CRD2, CRD3, and a transmembrane region. Gray boxes indicate different or absent amino acid. Red characters indicate an essential amino acid for EnvE recognition.

FIGURE S4 Trans-synaptic tracing using the oEnvE/oTVE system. **(A)** Monosynaptic tracing of layer 5 excitatory neurons in V1. Mixed AAVs were injected into the V1 of layer 5-specifc Tlx3-Cre mice: AAV2/9-CAG-DIO-H2B-mRuby3-IRES-oTVE-H and AAV2/9-CAG-DIO-oG. After the AAV injection, oEnvE-RVΔG-GFP were co-injected into the same location of the V1. **(B–C)** Typical images of the monosynaptically restricted tracing to the layer 5 neurons of V1. H2B-mRuby3-expressing neurons (nuclear-localized mRuby3) of the mouse V1 were specifically infected with oEnvE-RVΔG-GFP. GFP signals were enhanced by immunostaining. **(B)** Starter neurons and their presynaptic neurons in the V1. Closed arrowheads indicate H2B-mRuby3^+^/oEnvE-RVΔG-GFP^ +^ starter neurons. Scale bar: 100 μm. **(C)** Presynaptic neurons in the dLGN. Scale bar: 50 μm.

TABLE S1 Percentages of co-infected cells. Percentage of oEnvX-RVΔG-infected cells in oTVX-expressing cells in Figures 5C–F were shown. Data were obtained from three mice.
