# Supplementary figures and images for: Multiplex Neural Circuit Tracing With G-Deleted Rabies Viral Vectors
Source: Front Neural Circuits. 2020 Jan 10;13:77. doi: 10.3389/fncir.2019.00077 (PMC6967742; doi:10.3389/fncir.2019.00077)

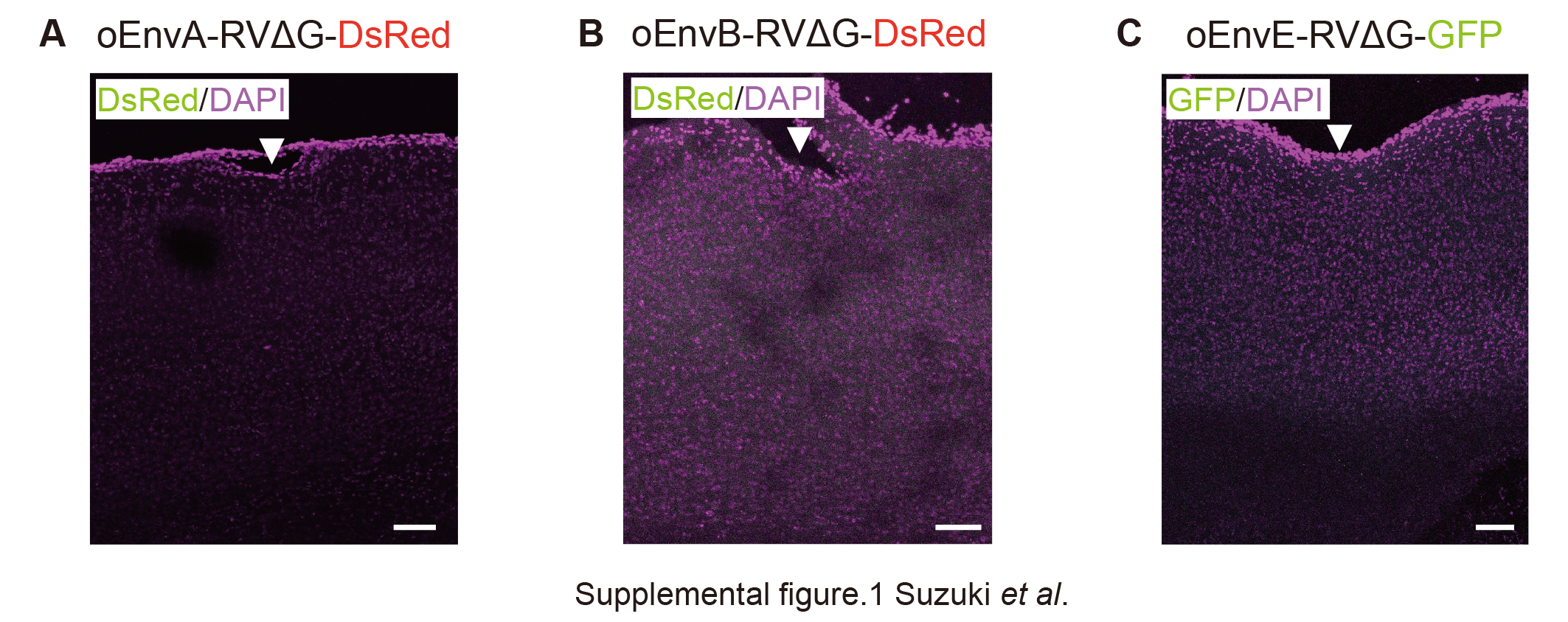

Supplement: FIGURE S1 — Control injection of oEnvX-RVΔG in the absence of oTVX. No cells were infected with oEnvA-RVΔG-DsRed (A), oEnvB-RVΔG-DsRed (B), or oEnvE-RVΔG-GFP (C) without injection of AAV expressing corresponding receptor oTVX. These results indicate no sign of contamination by unpseudotyped RVΔG in oEnvA-RVΔG-DsRed (A), oEnvB-RVΔG-DsRed (B), and oEnvE-RVΔG-GFP (C). Arrowheads indicate viral injection sites. Scale bar: 100 μm. [file Image_1.TIF]

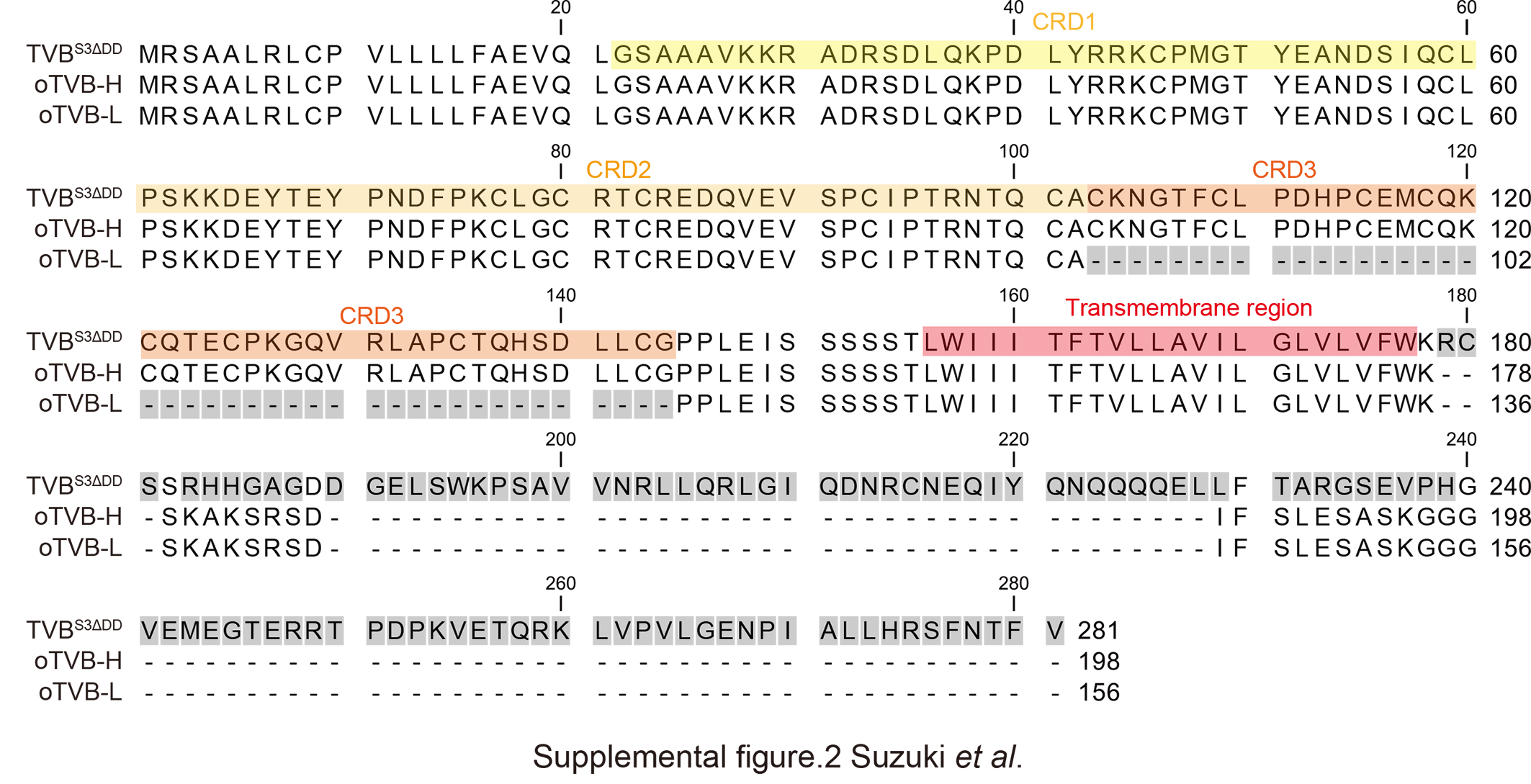

Supplement: FIGURE S2 — Protein sequence alignment for TVBS3, oTVB-H, and oTVB-L. Colored regions indicate functional domains: CRD1, CRD2, CRD3, and a transmembrane region. Gray boxes indicate absent or different amino acid. [file Image_2.TIF]

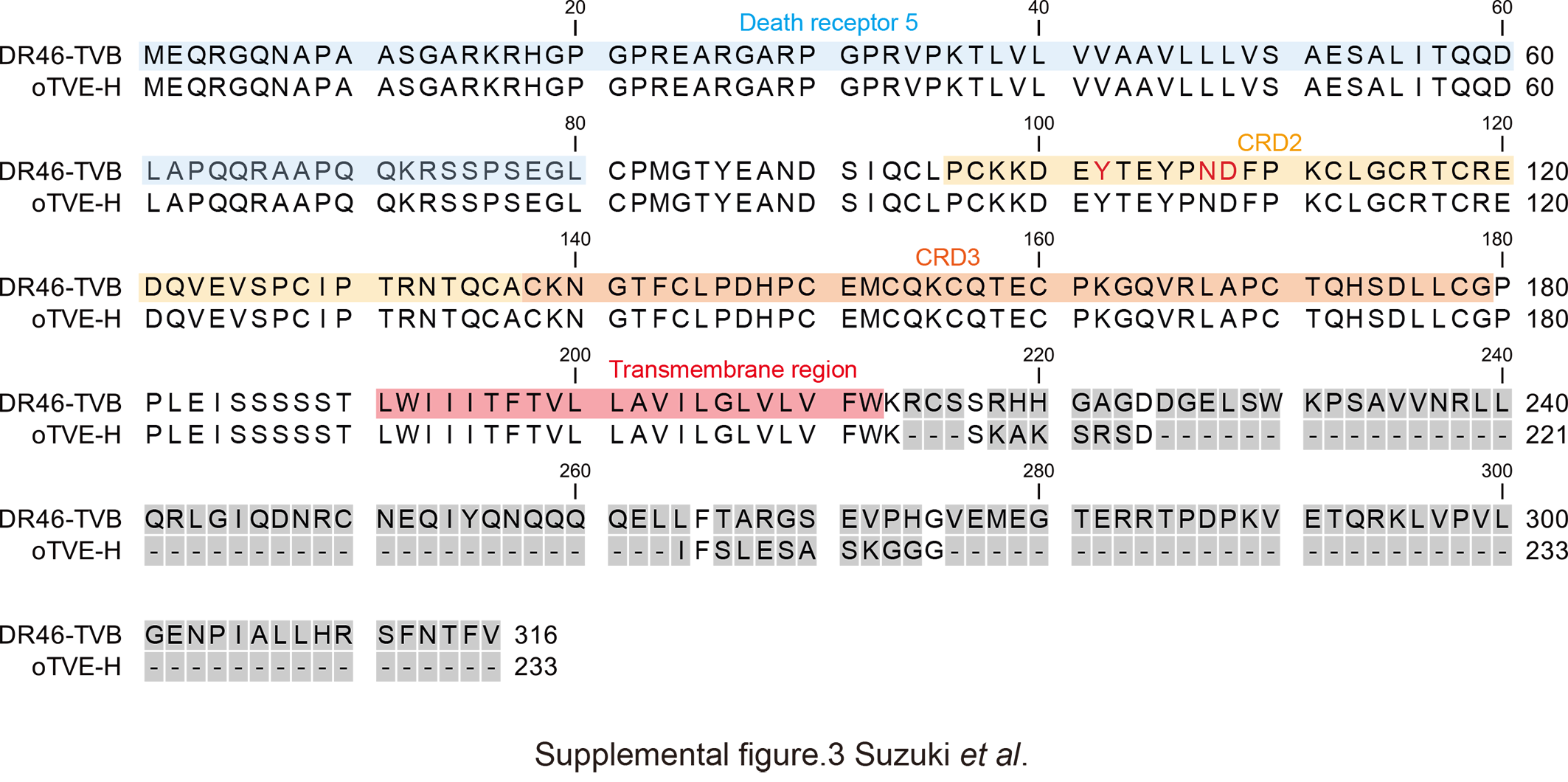

Supplement: FIGURE S3 — Protein sequence alignment for DR46-TVB and oTVE-H. Colored regions indicate the functional domains: death receptor 5, CRD2, CRD3, and a transmembrane region. Gray boxes indicate different or absent amino acid. Red characters indicate an essential amino acid for EnvE recognition. [file Image_3.TIF]

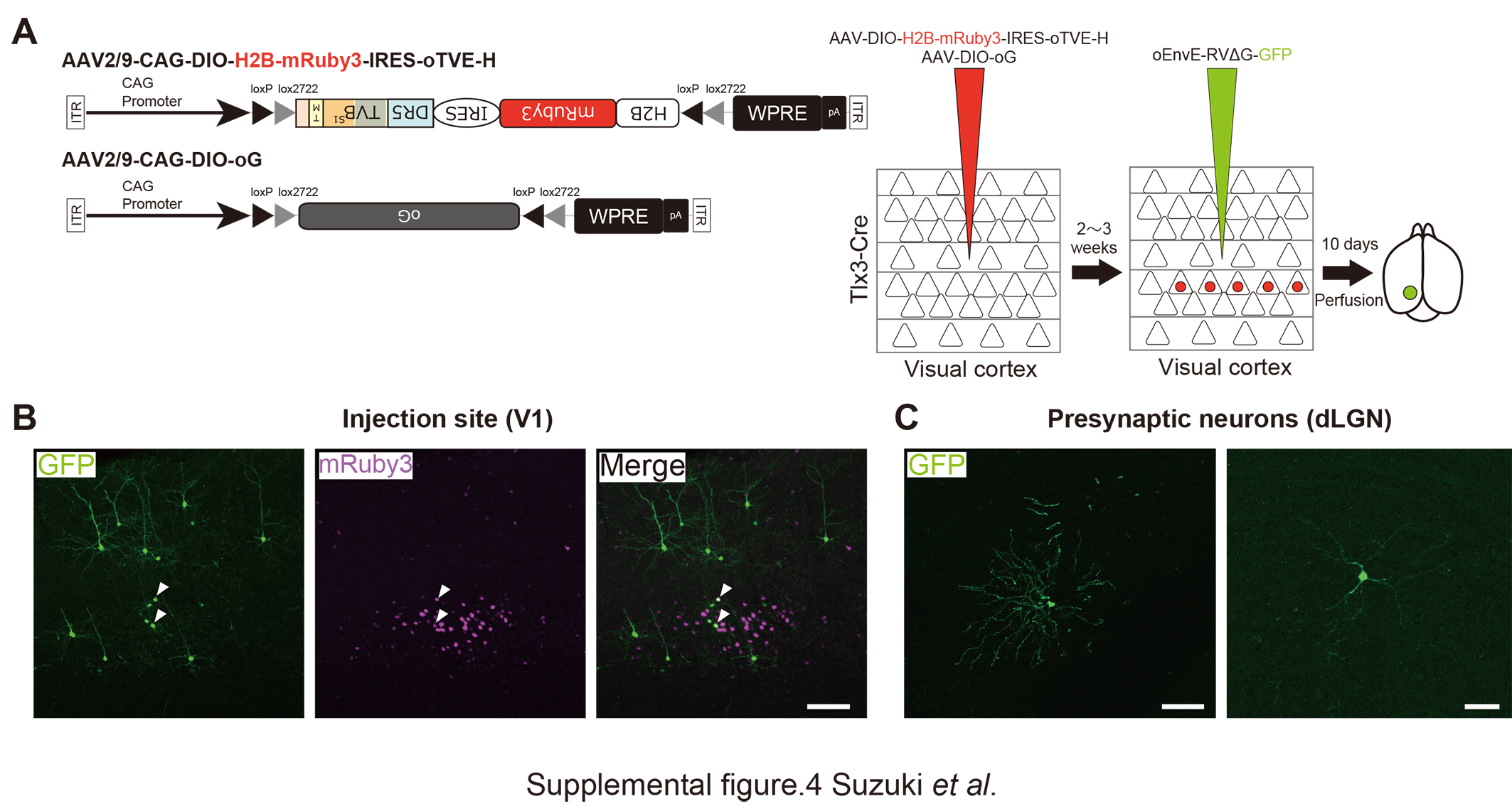

Supplement: FIGURE S4 — Trans-synaptic tracing using the oEnvE/oTVE system. (A) Monosynaptic tracing of layer 5 excitatory neurons in V1. Mixed AAVs were injected into the V1 of layer 5-specifc Tlx3-Cre mice: AAV2/9-CAG-DIO-H2B-mRuby3-IRES-oTVE-H and AAV2/9-CAG-DIO-oG. After the AAV injection, oEnvE-RVΔG-GFP were co-injected into the same location of the V1. (B–C) Typical images of the monosynaptically restricted tracing to the layer 5 neurons of V1. H2B-mRuby3-expressing neurons (nuclear-localized mRuby3) of the mouse V1 were specifically infected with oEnvE-RVΔG-GFP. GFP signals were enhanced by immunostaining. (B) Starter neurons and their presynaptic neurons in the V1. Closed arrowheads indicate H2B-mRuby3+/oEnvE-RVΔG-GFP + starter neurons. Scale bar: 100 μm. (C) Presynaptic neurons in the dLGN. Scale bar: 50 μm. [file Image_4.TIF]

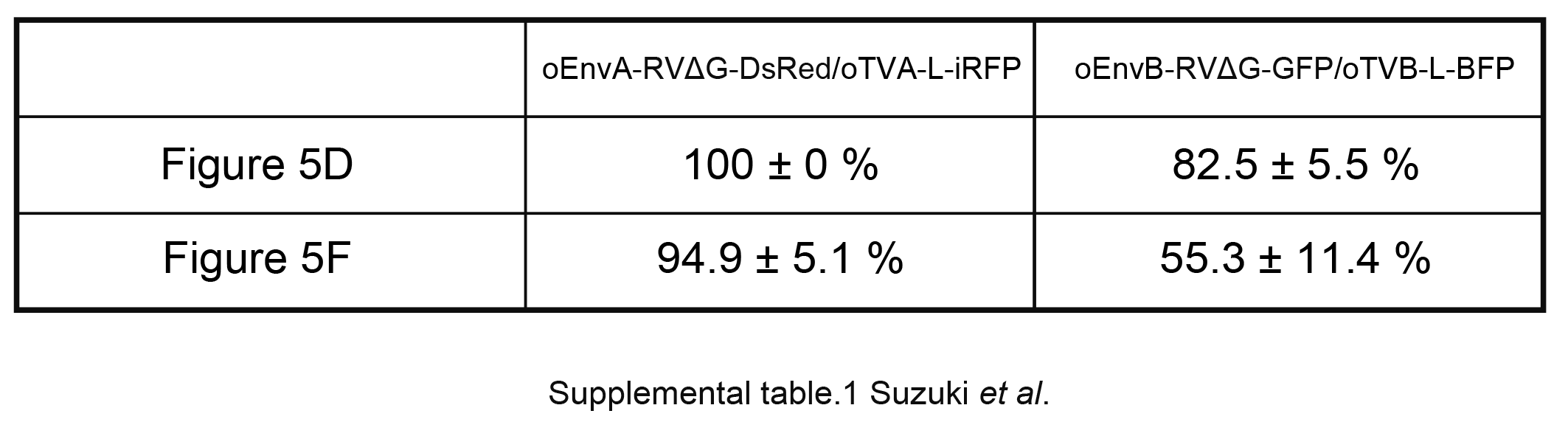

Supplement: TABLE S1 — Percentages of co-infected cells. Percentage of oEnvX-RVΔG-infected cells in oTVX-expressing cells in Figures 5C–F were shown. Data were obtained from three mice. [file Image_5.TIF]
